# Genome-wide Pleiotropy Analysis Reveals Shared Genetic Associations between Type 2 Diabetes Mellitus and Subcortical Brain Volumes

**DOI:** 10.34133/research.0688

**Published:** 2025-05-06

**Authors:** Qiyu Zhao, Jiayuan Xu, Ziqing Shi, Yang Zhang, Xin Du, Ying Zhai, Jinglei Xu, Feng Liu, Quan Zhang

**Affiliations:** Department of Radiology, Tianjin Key Lab of Functional Imaging & Tianjin Institute of Radiology, Tianjin Medical University General Hospital, Tianjin 300052, China.

## Abstract

Type 2 diabetes mellitus (T2DM), a prevalent metabolic disorder marked by insulin resistance and hyperglycemia, has been linked to volumetric changes in subcortical regions, yet the genetic basis of this relationship remains unclear. We analyzed genome-wide association study summary data for T2DM and 14 subcortical volumetric traits, using MiXeR to quantify shared genetic architecture and applying conditional/conjunctional false discovery rate analyses to detect novel and shared genomic loci. Enrichment and gene expression analyses were subsequently performed to explore the biological functions and mechanisms of genes associated with these loci. We observed a substantial proportion of trait-influencing variants shared between T2DM and subcortical structures, with Dice coefficients ranging from 22.4% to 49.6%. Additionally, 70 distinct loci were identified as being jointly associated with T2DM and subcortical volumes, 5 and 22 of which were novel for T2DM and subcortical volumes, respectively. The 769 protein-coding genes mapped to these shared loci are enriched in metabolic and neurodevelopmental pathways and exhibit specific developmental trajectories, with 117 genes showing expression levels linked to both T2DM and subcortical structures. This study uncovered polygenic overlap between T2DM and subcortical structures, deepening our comprehension of the genetic factors linking metabolic disorders and brain health.

## Introduction

Type 2 diabetes mellitus (T2DM) is a globally prevalent metabolic disorder primarily characterized by reduced insulin sensitivity and impaired glucose regulation [1]. In addition to its well-known metabolic complications, T2DM has been increasingly recognized for its impact on the central nervous system [2,3]. Epidemiological and neuroimaging studies have consistently demonstrated that T2DM increases the risk of cognitive impairment and dementia, which are associated with degenerative changes in brain structure [4–6]. Subcortical structures, such as the hippocampus, amygdala, caudate nucleus, and thalamus, are critical for brain functions like memory, emotional regulation, and motor control [7,8]. These regions are particularly susceptible to the effects of T2DM, highlighting their importance in understanding the neurobiological consequences of the disease.

Neuroimaging studies have revealed notable structural alterations in subcortical regions associated with T2DM. The hippocampus, crucial for memory formation and spatial navigation, has a reduced volume in individuals with T2DM [5,9,10]. Similarly, the amygdala, a key region for emotional processing, has exhibited structural changes in those affected by the disease [9–11]. The thalamus, which participates in transmitting sensory and motor signals, has also demonstrated structural abnormalities in T2DM patients [9,12]. Other subcortical structures, including the caudate nucleus and putamen—integral components of the basal ganglia responsible for motor and cognitive processes—have shown notable volume reductions in T2DM [5,9,12]. Furthermore, regions like the pallidum for movement regulation and the accumbens nucleus for reward processing are similarly affected [12,13]. These neuroimaging findings collectively highlight the close relationship between T2DM and subcortical structures, offering crucial insights into the neurobiological mechanisms underlying the disease.

Although subcortical abnormalities in T2DM have been widely reported, the molecular mechanisms driving these changes remain elusive. As a polygenic disorder, T2DM is influenced by numerous genetic variants distributed across the genome. A recent genome-wide association study (GWAS) has identified over 600 genetic loci associated with T2DM, reflecting the complex nature of its genetic architecture [14]. Similarly, subcortical brain structures are also shaped by a polygenic framework, with multiple genetic variants influencing their volume and morphology [15–17]. Given the high heritability of both T2DM and subcortical volumetric traits, it is plausible that overlapping genetic factors may explain the observed relationship between T2DM and subcortical abnormalities. Previous studies have indicated that specific genetic variants related to T2DM are involved not only in metabolic pathways but also in subcortical volumetric traits, such as *TCF7L2* linked to amygdala volume [18] and *Hp* 1-1 linked to hippocampal volume [19]. Moreover, polygenic risk scores (PRSs) for hemoglobin A1c, a crucial diagnostic marker for diabetes, have been associated with gray matter volume [20], and PRSs for multiple hippocampal shape features have been linked to diabetes [21]. Despite these advancements, the precise genetic architecture linking T2DM and subcortical brain volumes is still not well understood. Further research on identifying and characterizing the shared genetic variants is particularly crucial for advancing our understanding of the molecular pathways linking metabolic and brain health.

Traditional methods, such as PRS analysis, are limited in capturing specific genetic variants that contribute to complex traits and may underestimate the true extent of genetic overlap [22,23]. Advanced approaches, like the bivariate causal mixture model (MiXeR) [24,25] and conditional/conjunctional false discovery rate (cond/conjFDR) analyses [26,27], provide a more comprehensive framework for exploring genetic architecture. By integrating causal mixture models, MiXeR quantifies the genetic overlap between 2 traits, even when the genetic correlation is low or absent [25]. Additionally, cond/conjFDR analyses enhance the ability to discover new risk variants and detect shared genetic variants by leveraging data from multiple GWASs [26,27]. Together, these approaches provide more robust and precise tools for unraveling the genetic underpinnings of complex traits.

In this study, we aimed to investigate the polygenic overlap between T2DM and subcortical brain volumes. Using cutting-edge analytical techniques with large-scale GWAS summary data, we focused on mapping the global genetic architecture and identifying shared genomic variants. Specifically, MiXeR was employed to quantify the degree of polygenic overlap, and cond/conjFDR analyses were applied to detect novel loci associated with each trait and genomic loci shared between T2DM and subcortical brain volumes. Additionally, we performed functional annotation and enrichment analysis to clarify the biological roles and pathways underlying these genetic associations. The expression profiles of the identified shared genes were analyzed across different developmental periods, and their relevance to T2DM and subcortical brain structures was further explored. Through these comprehensive approaches, our study could provide a deeper understanding of the influence of T2DM on subcortical brain structures.

## Results

### Quantifying the polygenic overlap between T2DM and subcortical brain volumes

**Fig. 1.**
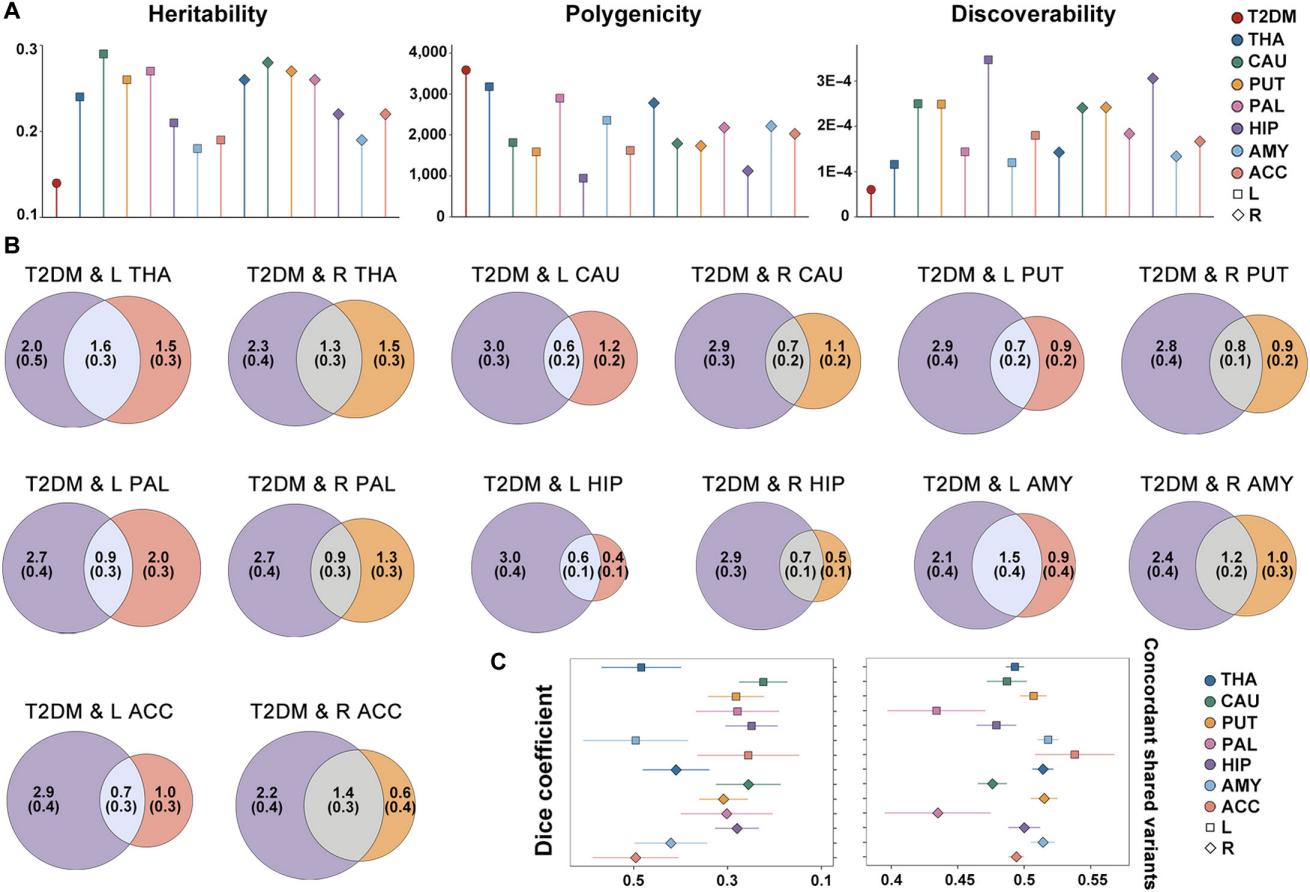
Polygenic overlap between type 2 diabetes mellitus (T2DM) and subcortical structures through MiXeR. (A) Univariate MiXeR assessed heritability, polygenicity, and discoverability for each trait. (B) Venn diagrams displaying the number of trait-specific and shared variants for T2DM and subcortical brain volumetric traits, as estimated by bivariate MiXeR. The number of trait-specific and shared trait-influencing variants are depicted in thousands with standard errors. The degree of polygenicity is reflected in the size of the circles. (C) The Dice coefficient and the proportion of concordant shared variants for each T2DM–volume pair. The error bars reflect the standard deviation. ACC, accumbens; AMY, amygdala; CAU, caudate; HIP, hippocampus; L, left; PAL, pallidum; PUT, putamen; R, right; THA, thalamus.

Univariate MiXeR [24] analysis characterized the heritability, polygenicity, and discoverability for T2DM and subcortical brain volumes. The findings showed a lower single-nucleotide polymorphism (SNP) heritability of 0.140, a higher polygenicity with 3,588 trait-influencing variants, and a lower discoverability of 6.05 × 10^−5^ for T2DM than for all subcortical brain volumes. Conversely, the subcortical brain volumes exhibited SNP heritability ranging from 0.182 to 0.295, polygenicity ranging from 947 to 3,177 trait-influencing variants, and discoverability ranging from 1.16 × 10^−4^ to 3.47 × 10^−4^ (Table S1 and Fig. 1A).

Bivariate MiXeR analysis [25] revealed substantial and varying number of shared variants between T2DM and each volumetric trait (Table S2, Fig. 1B, and Figs. S1 to S14). T2DM shared the highest proportion of its trait-influencing variants with the left thalamus (45.6%, 1,638 out of 3,588 variants) and the lowest proportion with the left hippocampus (15.7%, 564 out of 3,588 variants). In contrast, the right accumbens shared the highest proportion of its trait-influencing variants with T2DM (68.7%, 1,397 out of 2,032 variants) and the left pallidum shared the lowest proportion of its trait-influencing variants with T2DM (31.1%, 902 out of 2,897 variants) (Table S2 and Fig. 1B). The Dice coefficient, representing the proportion of shared trait-influencing variants among total variants, varied from 22.4% for T2DM and left caudate volume to 49.6% for T2DM and right accumbens volume (Table S2 and Fig. 1C). Moreover, the genetic influences of overlapping variants between T2DM and subcortical brain volumes exhibited mixed-effect patterns, with the proportion of consistent effects varying from 43.4% for T2DM and left pallidum to 53.8% for T2DM and left accumbens (Table S2 and Fig. 1C).

### Identifying novel and shared loci for T2DM and subcortical brain volumes

**Fig. 2.**
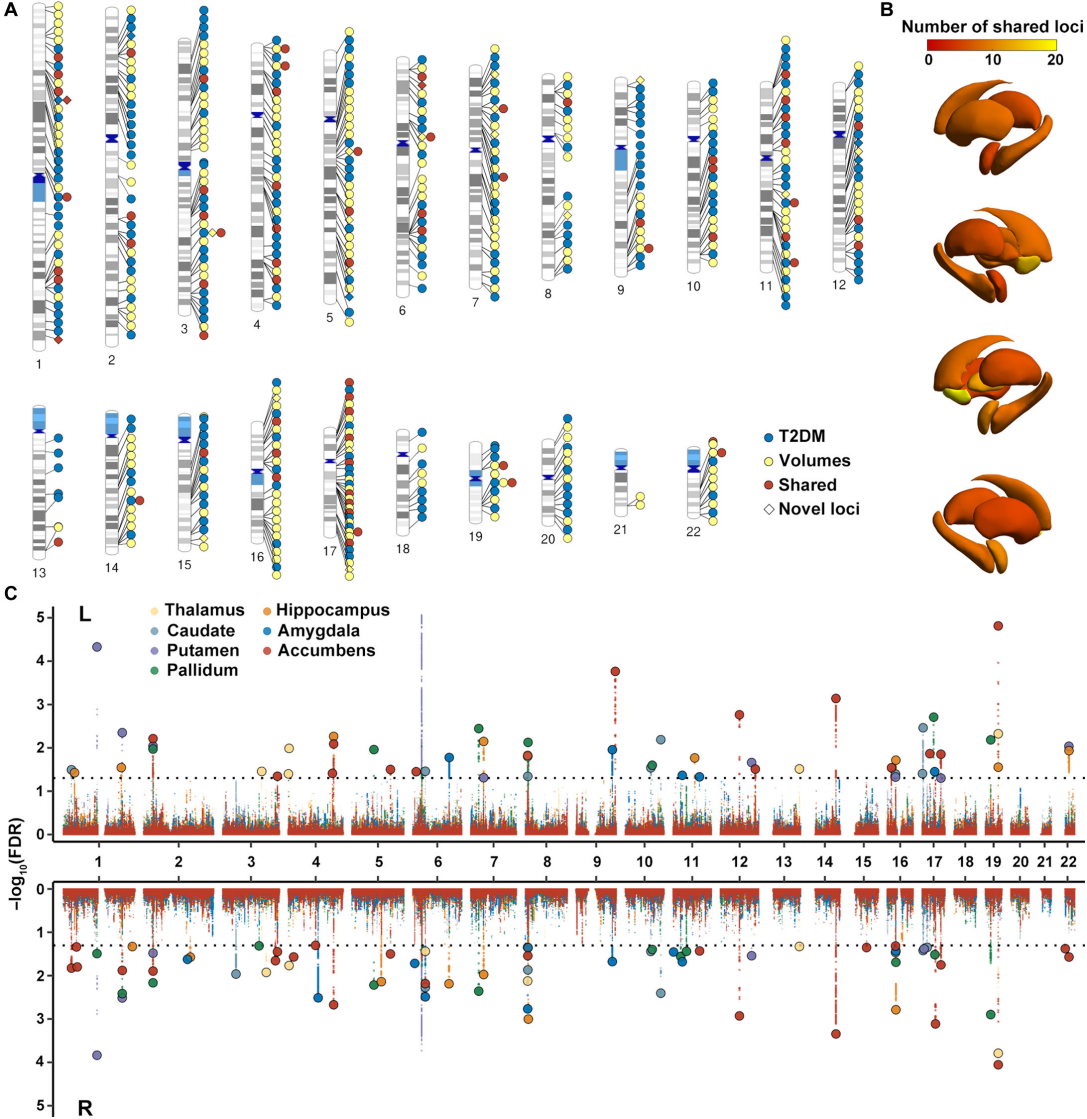
Results of genomic loci shared between T2DM and subcortical structures by conditional/conjunctional false discovery rate (cond/conjFDR) analyses. (A) Distribution of 229 distinct loci for T2DM, 220 distinct loci for subcortical volumes, and 70 distinct loci shared between the 2 traits by condFDR/conjFDR analyses, spanning across the genomic landscape of chromosomes. (B) Brain map displaying the locus count for each subcortical volume shared with T2DM. As the color shifts from red to yellow, it signifies a growing number of shared loci. (C) Manhattan plot demonstrating the relationships between T2DM and subcortical structures discovered through conjFDR. The top and bottom panels show the results of the left and right subcortical volumetric traits, respectively. Each point represents a single-nucleotide polymorphism (SNP), with larger points outlined in black indicating lead SNPs. The *y* axis shows −log_10_-transformed conjFDR values for each SNP, and the *x* axis indicates the chromosomal position. The threshold of conjFDR = 0.05 is represented by the dashed line, with different subcortical volumetric traits indicated by the color of the dots.

To improve the discovery of novel loci for each phenotype and shared loci between T2DM and subcortical brain volumes, condFDR and conjFDR analyses [26,27] were employed. The stratified quantile–quantile plots displayed enrichment of SNP associations for T2DM given subcortical brain volumes, and vice versa (Figs. S15 to S28). At condFDR < 0.01, we identified a total of 2,603 genomic risk loci related to T2DM by conditioning on the thalamus (left *N* = 191 and right *N* = 182), caudate (left *N* = 180 and right *N* = 180), putamen (left *N* = 183 and right *N* = 189), pallidum (left *N* = 184 and right *N* = 186), hippocampus (left *N* = 189 and right *N* = 183), amygdala (left *N* = 190 and right *N* = 186), and accumbens (left *N* = 189 and right *N* = 191) (Table S3 and Figs. S29 to S31). A combination of these loci across 14 subcortical volumetric traits revealed 229 distinct loci, with 5 of them being novel for T2DM, conditional on subcortical volumetric traits (Table S4 and Fig. 2A). Conditioning on SNP associations with T2DM, condFDR analysis revealed 399 loci across subcortical brain volumes, including the thalamus (left *N* = 13 and right *N* = 20), caudate (left *N* = 40 and right *N* = 44), putamen (left *N* = 43 and right *N* = 42), pallidum (left *N* = 22 and right *N* = 40), hippocampus (left *N* = 29 and right *N* = 28), amygdala (left *N* = 13 and right *N* = 12), and accumbens (left *N* = 27, right *N* = 26) (Table S5 and Figs. S32 to S34). Across all subcortical volumetric traits, we detected 220 distinct loci, 16 of which were newly discovered for subcortical brain volumes (Table S6 and Fig. 2A).

**Fig. 3.**
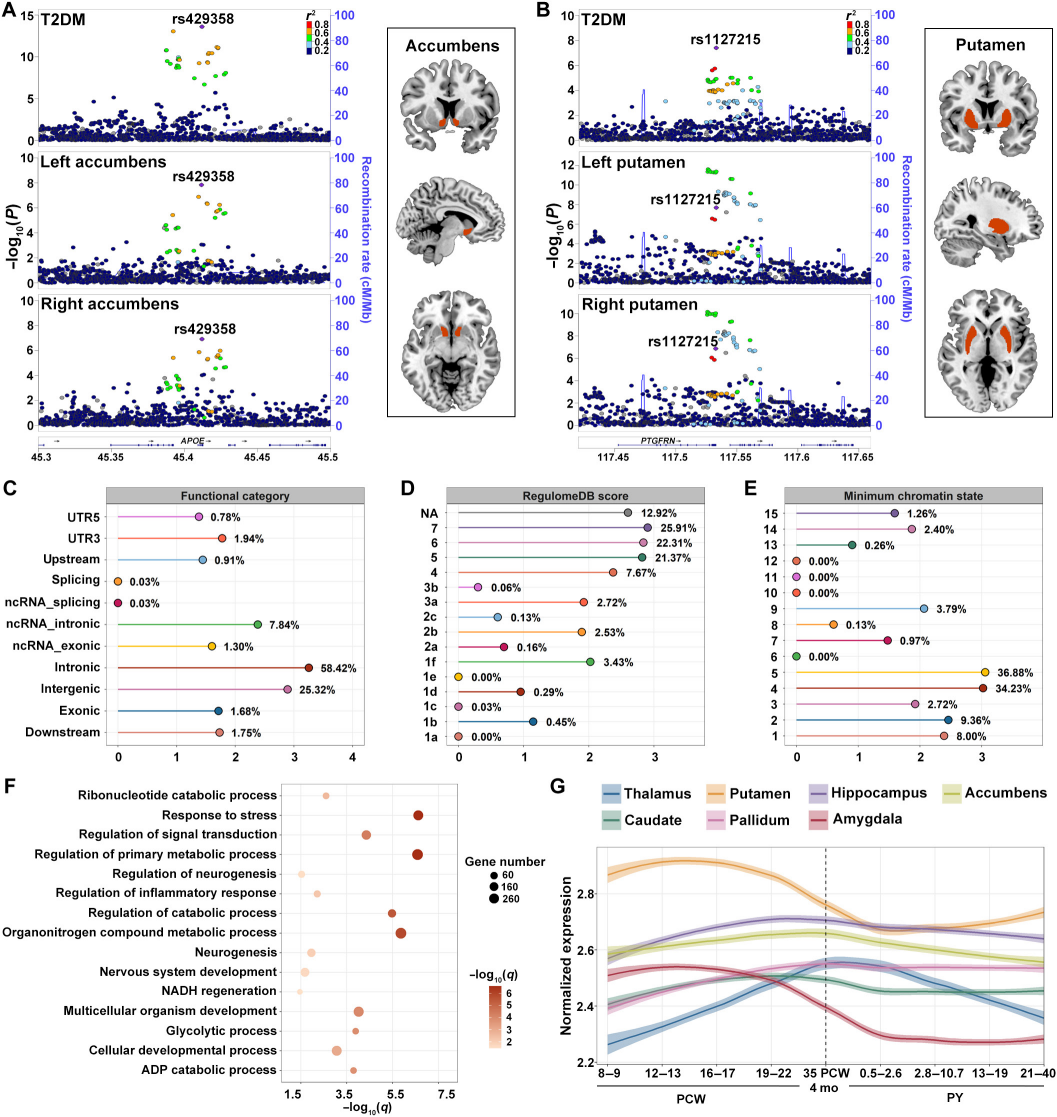
(A) LocusZoom plots of a genomic locus (chr19:45387596-45428234; top lead SNP: rs429358) showing regional SNP associations for T2DM and bilateral accumbens volumes. (B) LocusZoom plots of a genomic locus (chr1:117525810-117560929; top lead SNP: rs1127215) showing regional SNP associations for T2DM and bilateral putamen volumes. (C to E) Distribution of functional categories, RegulomeDB scores, and minimum chromatin states for all candidate SNPs within the shared loci associated with T2DM and subcortical structures. The *y* axis represents the categories used to classify candidate SNPs, and the *x* axis displays the log_10_-transformed counts of candidate SNPs in each category. In panel (D), “NA” denotes SNPs that were not annotated in the RegulomeDB database. (F) Bubble chart showing the enriched pathways of Gene Ontology for mapped genes. The *x* axis shows the −log_10_-transformed FDR *q* values, and the *y* axis displays the involved biological processes. Each bubble size represents the number of genes enriched in the biological process. The significance level is indicated by the color intensity of the bubbles, with darker colors representing higher significance. (G) Development trajectories of average gene expression for genes shared between T2DM and subcortical brain volumes. The *x* axis indicates 9 developmental stages, and the *y* axis presents the log_2_-transformed mean expression value. The shaded area indicates the 95% confidence interval, and the vertical dotted line represents the timing of births. ADP, adenosine diphosphate; NADH, reduced form of nicotinamide adenine dinucleotide; ncRNA, noncoding RNA; PCW, postconceptional weeks; PY, postnatal years; UTR3, 3′ untranslated region; UTR5, 5′ untranslated region.

At conjFDR < 0.05, a sum of 129 genetic loci were jointly associated with T2DM and subcortical brain volumes, including the thalamus (left *N* = 6 and right *N* = 6), caudate (left *N* = 8 and right *N* = 8), putamen (left *N* = 8 and right *N* = 5), pallidum (left *N* = 7 and right *N* = 12), hippocampus (left *N* = 8 and right *N* = 8), amygdala (left *N* = 5 and right *N* = 10), and accumbens (left *N* = 15 and right *N* = 23) (Table S7 and Fig. 2A to C). Among the subcortical brain regions, the top 2 significant loci were chr19:45387596-45428234 for T2DM and volume of the bilateral accumbens (lead SNP: rs429358; left: conjFDR = 1.54 × 10^−5^; right: conjFDR = 8.79 × 10^−5^; Table S7 and Fig. 3A) and chr1:117525810-117560929 for T2DM and volume of the bilateral putamen (lead SNP: rs1127215; left: conjFDR = 4.69 × 10^−5^; right: conjFDR = 1.46 × 10^−4^; Table S7 and Fig. 3B). These 2 lead SNPs, rs429358 and rs1127215, were located in open chromatin state regions with a minimum chromatin state below 7. Additionally, rs429358, a nonsynonymous exonic variant, was annotated to *APOE* as the nearest gene and had a combined annotation-dependent depletion (CADD) score higher than 12.37, indicating a high level of deleterious effect. Next, by examining the impact directions of the lead SNPs at shared loci, we observed that 40% (52/129) were consistent between T2DM and subcortical brain volumes, and 60% (77/129) had opposite directions (Table S7). Furthermore, when combining these loci across all T2DM–volume pairs, 29 genomic loci were linked to T2DM and at least 2 subcortical brain structures (Table S8 and Fig. S35), yielding 70 distinct shared loci (Table S9). Among these distinct loci, 5 were newly identified for T2DM, 22 were newly discovered for subcortical brain volumes, and 3 were novel for both traits (Table S9).

The functional annotation [28] of all candidate SNPs (*N* = 3,088) at conjFDR < 0.1 for T2DM and subcortical brain volumes revealed that a large proportion were positioned in intronic (58%, 1,804/3,088) and intergenic (25%, 782/3,088) regions (Table S10 and Fig. 3C). Additionally, a total of 25 nonsynonymous exonic variants were uncovered (Table S10). Among the shared loci, 4.7% (147/3,088) of the candidate SNPs displayed a CADD score above 12.37, suggesting potential deleterious effects. Approximately 4.2% (130/3,088) of the candidate SNPs had RegulomeDB scores less than 2, implying a possible influence on transcription factor binding (Fig. 3D). Additionally, 92.2% (2,846/3,088) of the candidate SNPs were found within open chromatin state regions, with the distribution of minimum chromatin state ranging from 1 to 7 (Fig. 3E).

Using 3-way gene-mapping strategies, all candidate SNPs within the shared genomic loci were linked to 769 protein-coding genes (Table S11). All genes annotated to candidate SNPs exhibited significantly down-regulated differentially expressed genes in 3 of 30 tissues, specifically in the pancreas, liver, and heart (Fig. S36). As shown in Table S12, gene-set enrichment from the GWAS catalog was observed in 144 traits, mainly including brain morphology (FDR *q* = 1.88 × 10^−53^), body fat distribution (FDR *q* = 2.23 × 10^−21^), and type 2 diabetes (FDR *q* = 1.33 × 10^−18^). Furthermore, enrichment analysis identified 501 significant Gene Ontology (GO) biological process terms after correction for multiple comparisons, primarily including the regulation of primary metabolic process (FDR *q* = 2.59 × 10^−7^), positive regulation of cellular metabolic process (FDR *q* = 9.46 × 10^−7^), nervous system development (FDR *q* = 2.10 × 10^−2^), and regulation of neurogenesis (FDR *q* = 2.95 × 10^−2^) (Table S13 and Fig. 3F).

### Distinct spatiotemporal expression trajectories of shared genes

Utilizing spatiotemporal brain expression trajectory analysis with PsychENCODE [29], we explored the developmental patterns of genes that are jointly associated with T2DM and subcortical structures throughout the lifespan. As depicted in Fig. 3G, the genes shared between T2DM and the putamen and amygdala exhibit comparable patterns, characterized by elevated expression during the fetal period, peaking around 12 to 17 postconceptional weeks (PCW), followed by a gradual decline. The lowest expression levels are observed in the postnatal period (0.5 to 2.6 postnatal years [PY]), with a modest increase during adulthood. The caudate and pallidum exhibit a steady increase in gene expression during the fetal period, followed by a slight decline after 35 PCW and eventual stabilization in the postnatal period. For the hippocampus and accumbens, gene expression gradually rises throughout the fetal period, with a minor decrease after 35 PCW and no substantial fluctuations after that. The thalamus demonstrates a consistent increase in expression during the fetal period, with a distinct turning point at 35 PCW, after which expression steadily declines, exhibiting marked variability throughout later development.

### Expression–trait associations

**Fig. 4.**
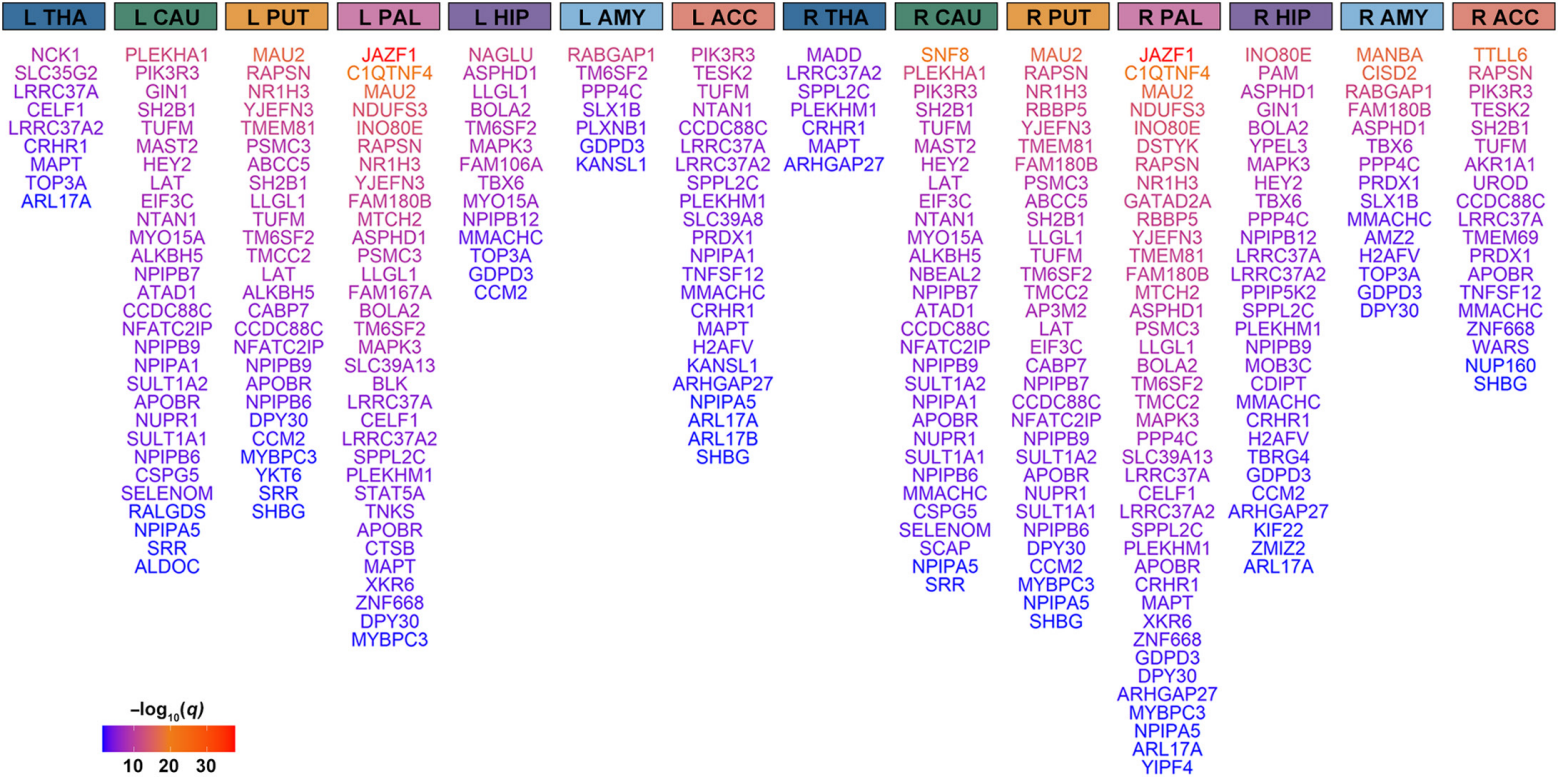
MetaXcan identifies 117 unique genes jointly associated with T2DM and subcortical brain volumes. The plot shows all genes with significant expression–trait associations for T2DM and at least one subcortical brain volume. Each column shows genes related to both T2DM and the corresponding subcortical volume shown above. The color represents the −log_10_-transformed FDR-adjusted *q* values of the expression–T2DM associations. The transition from blue to red reflects the increasing significance of the association with T2DM, and higher gene positions indicate a smaller *q* value.

We applied the MetaXcan tool [30] to explore the associations between brain gene expression and the subcortical volumetric traits and their associations with T2DM. Of the shared genes, 384 significant associations were observed between gene expression and subcortical brain volumes, corresponding to 154 shared genes (Table S14). Among these 154 genes, 117 genes were also discovered to have a correlation with T2DM (Table S15 and Fig. 4). In the analysis of expression–volume associations, *TUFM* exhibited the strongest correlation with the right caudate volume, followed by *CCDC88C*, which was significantly associated with the right accumbens volume. These 2 genes were also significantly linked to the volumes of the bilateral caudate, putamen, accumbens, and T2DM. The strongest associations between gene expression and T2DM were found for *JAZF1* and *C1QTNF4*, which were also correlated with the bilateral pallidum volumes.

### Validating analysis

To validate the loci shared between T2DM and subcortical brain structures, we conducted local genetic correlation analyses using local analysis of [co]variant association (LAVA) [31]. Univariate analyses identified 70 out of the 129 shared loci with nominal significance for local heritability in both T2DM and subcortical brain volumes (*P* < 0.05, Table S7). Among the 70 shared loci, bivariate analyses revealed 29 with significant local genetic correlations (FDR *q* < 0.05), including 11 showing positive correlations between 0.52 and 1.00 and 18 displaying negative correlations between −0.40 and −1.00 (Table S7). The strongest positive correlation was found at chr5:76424949-76435346 between T2DM and the bilateral pallidum (lead SNP: rs6878122; right: *r* = 1.00, *q* = 7.59 × 10^−3^, conjFDR = 6.06 × 10^−3^; left: *r* = 0.76, *q* = 1.97 × 10^−2^, conjFDR = 1.10 × 10^−2^). The strongest negative correlation was detected at chr1:201760981-201886402 between T2DM and the left hippocampus (lead SNP: rs7551784; *r* = −0.99, *q* = 1.54 × 10^−3^, conjFDR = 2.85 × 10^−2^).

To assess the replication of shared genomic loci between T2DM and subcortical volumetric traits, we further examined the consistency of allelic effect directions using independent datasets. In the T2DM dataset, 103 out of 129 top lead SNPs were observed in the independent dataset, with 78 of these SNPs exhibiting consistent effect directions (*P* = 8.05 × 10^−8^). For the 14 subcortical volumetric phenotypes, 110 top lead SNPs were identified in the independent dataset, with 67 SNPs showing consistent effect directions (*P* = 1.39 × 10^−2^). These findings reinforce the robustness of the shared genetic loci between T2DM and subcortical structural changes, with consistent allelic effects across different ancestries supporting the stability of the underlying genetic architecture.

## Discussion

In this study, we performed a series of genetically informed analyses to explore the relationship between T2DM and subcortical brain volumes. Our findings provide evidence of polygenic overlap between these traits, demonstrating the genetic connection between diabetes and brain structure. Using the MiXeR tool [25], we quantified substantial genetic overlap, with Dice coefficients ranging from 22.4% to 49.6%, reflecting differences in the genetic architectures of T2DM and subcortical volumetric traits. Through cond/conjFDR approaches [26], we identified 129 genomic loci jointly associated with the 2 phenotypes, with 70 distinct shared loci across all T2DM–volume pairs. Among the 70 loci, 5 were newly identified for T2DM and 22 were newly identified for subcortical brain volumes. Subsequent enrichment and gene expression analysis uncovered the prospective biological roles of the shared genes and their expression patterns in brain tissues. These findings strengthen the understanding of the link between T2DM and subcortical structures, providing valuable insights into the underlying mechanisms involved.

Although it is well established that T2DM causes alterations in subcortical morphology compared with healthy controls [9–11], the genetic basis for these changes remains unknown. Univariate MiXeR analysis showed that T2DM, with 3,588 trait-influencing variants, exhibits higher polygenicity compared to subcortical brain volumes (947 to 3,177 trait-influencing variants), indicating a more complex genetic architecture for T2DM involving a broader spectrum of trait-influencing variants. However, T2DM (discoverability of 6.05 × 10^−5^) is less genetically discoverable than subcortical volumetric traits (discoverability ranging from 1.16 × 10^−4^ to 3.47 × 10^−4^), suggesting that a larger sample size is necessary to fully reveal the genetic architecture of T2DM compared to that for subcortical brain volumes. Moreover, bivariate MiXeR analysis demonstrated a substantial proportion of trait-influencing variants shared between T2DM and subcortical structures, although the extent of overlap varied across different subcortical brain regions. In cases of extensive genomic loci overlap, a mixture of agonistic and antagonistic effects likely occurs among shared variants [32], aligning with the observed concordance rates of around 50% between T2DM and subcortical volumes. These findings implicate multifaceted shared molecular biological processes between T2DM and subcortical volumes, with mixed-effect directions potentially involving both protective and detrimental mechanisms.

The condFDR analysis revealed numerous novel loci associated with T2DM and subcortical volumes, demonstrating the effectiveness of this method in identifying previously unknown genetic variants. Specifically, 5 novel loci were discovered for T2DM when conditioned on subcortical volumes, and 16 were newly identified for subcortical volumes when conditioned on T2DM. These findings suggest a potential interaction between metabolic and neurostructural pathways [33]. The conjFDR analysis offers a deeper understanding of the shared genetic mechanisms between T2DM and subcortical volumetric traits. Among the 129 jointly associated loci, the most significant was observed at the 19q13.32 region, with the lead SNP rs429358—a nonsynonymous mutation located in the exon of *APOE* gene—showing the strongest genetic link to changes in the volume of the accumbens. As a central component of the brain’s reward system, the accumbens plays a critical role in emotional regulation, motivation, and reward processing [34]. Consequently, changes in its volume may influence reward-related behaviors and emotional responses [35]. Metabolic dysregulation in T2DM, particularly insulin resistance and chronic low-grade inflammation, may affect the structure of the accumbens, thereby altering these behaviors and responses [36]. *APOE* is well-known for its role in neurodegenerative diseases, particularly Alzheimer disease, with the ε4 allele playing a key part in processes such as neuronal maintenance, neuroinflammation, lipid metabolism, and amyloid-β clearance [37–39]. The identification of this region suggested that *APOE* may contribute to structural brain changes by exacerbating metabolic imbalances, particularly in regions like the accumbens, which are linked to reward and emotional regulation [40]. These findings support the idea that T2DM may impact brain structures through metabolic pathways, particularly involving processes like neuroinflammation [41] and dysregulated lipid metabolism [42], potentially leading to alterations in subcortical volumes. Furthermore, as a nonsynonymous variant, the high CADD score for rs429358 supports its pathogenic potential, highlighting its significance in influencing gene function and phenotypic outcomes. Additionally, our study identified significant shared loci between T2DM and the volume of the putamen, located at the 1p13.1 region, with rs1127215 as the lead SNP. The putamen, a key structure within the basal ganglia, is involved in motor control, reward processing, and various aspects of learning and memory [43]. Dysregulation in this region has been closely linked to metabolic diseases, and T2DM may influence putamen volume, thereby affecting motor control and reward mechanisms [44]. This alteration may arise from metabolic disturbances, particularly dysregulated lipid metabolism and the impact of insulin resistance on neural plasticity [45]. The 1p13.1 region is located in *PTGFRN* gene, which regulates the prostaglandin F2 receptor and has been linked to lipid accumulation and T2DM [14]. These regions are located in open chromatin regions, indicating heightened transcriptional activity and suggesting regulatory roles in the interplay between T2DM and subcortical structural changes.

Our analysis of the effect directions for the shared loci revealed a complex and multifaceted pattern. Approximately 40% of the loci exhibited consistent effect directions between T2DM and subcortical volumetric traits, while the remining 60% showed opposite directions. This divergence underscores the intricate biological interplay between metabolic dysfunction and brain structure. Loci with consistent effect directions may indicate protective genetic mechanisms, whereby increased T2DM risk is associated with larger subcortical brain volumes [46]. These variants may influence metabolic pathways that preserve neuronal integrity, enhance synaptic plasticity, or mitigate neurodegenerative processes, thereby buffering the brain against the harmful effects of T2DM-related metabolic disturbances. In contrast, loci exhibiting opposing effect directions suggest detrimental mechanisms, linking higher T2DM risk to reduced subcortical brain volumes. These genetic variants may contribute to neurodegeneration, chronic neuroinflammation, or microvascular dysfunction, all of which are known contributors to brain atrophy in the context of T2DM [47]. Together, these findings highlight the heterogeneous nature of the genetic architecture connecting metabolic health and brain structure, with some variants exacerbating structural vulnerability, while others confer resilience. Additionally, a total of 29 shared loci were linked to at least 2 subcortical brain regions, suggesting pleiotropic effects across multiple structures. Among these, the locus at 8:8118600-11828200 showed the most widespread associations, being linked to 9 subcortical volumetric phenotypes (Table S8). This locus affects several key brain regions—including the thalamus, caudate, pallidum, accumbens, hippocampus, and amygdala—which are critically involved in emotional regulation, cognitive processing, reward signaling, and motor control [48]. The broad pattern of associations observed for this locus suggests that it may contribute to shared neurobiological pathways underlying both subcortical structural variation and the pathophysiology of T2DM.

Among the shared loci, the variant with the highest CADD score is rs62618693, located at 11p13, a nonsynonymous exonic variant, and has been identified as a causal SNP for T2DM [49]. This variant encodes the *QSER1* gene, which has been implicated as a pathogenic gene for T2DM [50]. *QSER1* is a DNA-binding protein that inhibits the activity of the DNA methyltransferases DNMT3A and DNMT3B, preventing excessive DNA methylation during development [51]. Disruption of *QSER1* function may impair the development and function of pancreatic β cells, increasing the risk of T2DM [50]. In our functional annotation of all candidate SNPs, 25 nonsynonymous exonic variants were identified. These variants directly alter the amino acid sequence of proteins, affecting their structure and function, which plays a crucial role in metabolic and neurostructural processes. Through 3-way gene-mapping strategies, the candidate SNPs were linked to 769 protein-coding genes. Enrichment analysis revealed significant GO biological processes, primarily involving positive regulation of cellular metabolic processes, cellular catabolic processes, and cellular response to stress, indicating a potential role in disrupting energy metabolism at the cellular level, with possible effects on neurons and glial cells. These disruptions may drive structural brain changes in T2DM through mechanisms like metabolic imbalance, inflammation, and oxidative stress [52], aligning with the characteristic metabolic dysregulation of T2DM and suggesting a close connection between metabolic abnormalities and compromised brain health [53,54]. Additionally, the enrichment of neurogenesis and nervous system development pathways suggested that T2DM-associated loci may influence early brain development, particularly in subcortical regions. These findings support the idea that T2DM affects not only metabolism but also neurodevelopment, potentially altering synaptic plasticity and neuron survival [55,56].

In the MetaXcan analysis, we explored the associations of brain gene expression with both subcortical volumetric traits and T2DM. Our findings identified that 117 genes were simultaneously correlated with T2DM and at least one subcortical brain structure. For instance, the expression levels of *TUFM* and *CCDC88C* were related to the volumes of the bilateral caudate, putamen, accumbens, and T2DM. *TUFM*, a mitochondrial translation elongation factor involved in protein synthesis and metabolic regulation, has been linked to diabetic cardiomyopathy [57]. *CCDC88C*, associated with neurodevelopment, may be important in regulating neural structures [58]. Additionally, the expression of *JAZF1* and *C1QTNF4* was linked to the volumes of the bilateral pallidum and T2DM. *JAZF1*, a key gene in metabolic regulation, has previously been linked to an increased susceptibility to T2DM [59]. *C1QTNF4*, a gene specifically expressed in the brain [60], is likely involved in the development of Alzheimer disease [61]. These complex relationships suggest that shared genes regulate metabolic and neurological pathways, playing multiple roles in the interplay between T2DM and brain structural changes, emphasizing their critical involvement in various biological processes.

The following limitations should be considered when interpreting our findings. First, the genomic association data used in this study originate from European populations, potentially limiting the generalizability of our results. Genetic diversity among different ethnic groups may affect the applicability of these genetic loci to other populations, and future research should validate these results in more diverse ethnic backgrounds. Second, our reliance on GWAS summary data may limit the ability to capture the influence of rare genetic variants on T2DM and subcortical brain volumes. Rare variants can play an important role in complex traits, but our analysis did not thoroughly investigate their potential contributions. Third, despite the use of advanced statistical models such as MiXeR and cond/conjFDR, these approaches have inherent limitations. Unmeasured environmental factors that may influence T2DM and brain structure may not be accounted for, potentially affecting the accuracy of our findings. Fourth, while enrichment and gene expression analyses provide insights into potential biological mechanisms, these are based on computational predictions. Experimental validation, such as studies in cellular or animal models, is essential to confirm the specific roles of the identified genetic loci.

In conclusion, our study confirmed the complex genetic relationship between T2DM and subcortical brain volumes, revealing the shared genetic architecture and providing new insights into the biological pathways linking metabolic and neurological processes. Through advanced statistical models and functional annotations, we revealed how genetic variants associated with T2DM may influence subcortical brain structures via mechanisms such as metabolic dysregulation, neuroinflammation, and impaired neurogenesis. These findings not only deepen our understanding of the genetic connections between metabolic disorders and brain health but also highlight the potential for targeted approaches in disease prevention and treatment, particularly through early intervention strategies aimed at mitigating the impact of T2DM on subcortical brain structures.

## Methods

### GWAS samples

For the subcortical volumetric traits, we acquired GWAS summary statistics for 14 subcortical regions, including the volumes of the bilateral thalamus, caudate, putamen, pallidum, hippocampus, amygdala, and accumbens. These data were sourced from the Oxford Brain Imaging Genetics (BIG40) web server, based on a sample of 33,224 UK Biobank participants of European ancestry [17]. GWAS summary statistics for T2DM were obtained from the DIAGRAM consortium, which involved a large multi-ancestry meta-analysis involving 428,452 cases and 2,107,149 controls [14]. For the MiXeR analysis, we chose the European ancestry subset of T2DM GWAS summary statistics, which included the largest available sample of European ancestry (242,283 cases and 1,569,734 controls, including UK Biobank participants) [14]. This choice was made because MiXeR can adjust for sample overlap, and the larger sample size enables a more accurate estimation of the genetic overlap between subcortical volumetric traits and T2DM while maximizing statistical power [25]. In contrast, for the cond/conjFDR method, which requires independent datasets to avoid inflated trait associations due to sample overlap [62,63], we used additional T2DM summary statistics excluding UK Biobank participants (55,005 cases and 400,308 controls) [64].

### Statistical analyses

#### MiXeR

We employed MiXeR v1.3 to quantify the polygenic overlap between T2DM and subcortical brain volumes [24,25]. MiXeR utilizes GWAS summary statistics to estimate the total number of trait-influencing variants and SNP heritability for each trait. This method relies on a detailed linkage disequilibrium (LD) structure modeled from European ancestry reference panel of 11 million SNPs [65] to accurately capture the genetic architecture of each trait [24]. To this end, we first conducted univariate MiXeR analysis to assess the polygenicity, discoverability, and heritability of each phenotype. Polygenicity refers to the quantity of trait-influencing variants required to explain 90% of the SNP heritability of a given trait. Traits displaying higher polygenicity are influenced by a greater number of trait-influencing variants compared to those exhibiting lower polygenicity. Discoverability is defined as the average strength of additive genetic effects among these trait-influencing variants. Traits with higher genetic discoverability have associated variants with larger average effect sizes, making these variants easier to detect. Heritability is calculated based on the combined effects of polygenicity and discoverability, representing the proportion of phenotypic variance in a population that can be attributed to genetic variation. Traits with higher heritability have a greater proportion of their variance explained by genetic differences.

Building on the univariate causal mixture model, bivariate MiXeR extends the analysis by modeling the genetic effects of each trait as a mixture of 4 bivariate normal distributions [25]. Two of these distributions represent variants specific to each phenotype, one represents variants influencing both phenotypes, and the final null distribution represents variants with no effect on either phenotype. Bivariate MiXeR estimates the number of shared and trait-specific variants that influence traits and the percentage of shared variants exhibiting the same effect direction between 2 traits. The results are visualized through Venn diagrams, illustrating the proportions of trait-specific and shared trait-influencing variants. Additionally, the Dice coefficient, measured by bivariate MiXeR, reflects the proportion of shared trait-influencing variants between the T2DM and subcortical volumes, offering a quantitative assessment of the similarity in their genetic architectures. To prevent potential bias caused by intricate LD structures, SNPs located in the major histocompatibility complex region (chr6:25119106-33854733) were removed from the analysis.

#### Cond/conjFDR analyses

Cond/conjFDR analyses were employed to enhance the identification of loci related to each trait and to detect loci shared between T2DM and subcortical brain volumes [26,27]. The condFDR method, built upon an empirical Bayesian statistical framework, utilizes the combined power of 2 GWAS data to improve the identification of genomic variants. This approach refines the selection of SNPs by considering their associations with the primary and secondary traits, identifying variants that are more likely to represent true associations despite not meeting genome-wide significance thresholds. For each trait pair, condFDR re-ranks the test statistics of the primary trait (e.g., T2DM) through adjusting for the SNP associations with the secondary trait (e.g., one certain subcortical volume). The cross-trait enrichment was assessed by constructing conditional quantile–quantile plots. In these plots, SNP associations with the primary trait were stratified according to their significance in the secondary trait, with *P* value thresholds set at <0.100, <0.010, and <0.001. Cross-trait enrichment was demonstrated by leftward deflection from the expected line as the strength of association in the secondary trait increased, reflecting enrichment of shared genetic loci between T2DM and subcortical structures. The conjFDR approach, an extension of condFDR, involves performing 2 condFDR analyses—conditioning subcortical volumes on T2DM and vice versa. The conjFDR value is defined as the maximum of the 2 corresponding condFDR values, providing a conservative estimate of the FDR for loci associated with both traits. Consistent with previous studies [66–69], the significance levels for condFDR and conjFDR analyses were established at 0.01 and 0.05, respectively. Before fitting the FDR model, we excluded SNPs located in the major histocompatibility complex (chr6:25119106-33854733) and 8p23.1 regions (chr8:7200000-12500000) to prevent bias arising from the complex LD patterns [70–72].

### Genomic loci definition

Following the FUMA [28] protocol, we identified the independent genomic loci. Independent significant SNPs were first identified based on condFDR < 0.01 or conjFDR < 0.05, with the requirement that these SNPs are independent of each other at *r*^2^ < 0.6. Among these SNPs, those in LD with *r*^2^ < 0.1 were selected as lead SNPs. Candidate SNPs were identified as those with condFDR/conjFDR < 0.10 and LD *r*^2^ > 0.6 with at least one independent significant SNP, and the boundaries of each locus were established by including all such candidate SNPs. Loci located within a 250-kb distance from each other were combined into a unified genomic locus. LD information for the analysis was obtained from the European ancestry reference panel of the 1000 Genomes Project [65].

We further identified distinct shared genomic loci spanning all T2DM–volume pairs. If 2 or more loci from different T2DM–volume pairs had genomic boundaries within 250 kb from each other, they were consolidated into a single distinct shared locus by combining their genomic boundaries. The lead SNP with the most significant FDR value was selected as the final lead SNP for the combined locus. A locus was deemed novel if it spanned more than 500 kb from the boundaries of any loci previously identified in the initial GWAS data (*P* < 1.0 × 10^−6^) [17,64] and findings from previous GWAS and cond/conjFDR studies (Tables S16 and S17) and if none of the candidate SNPs had been documented in the National Human Genome Research Institute–European Bioinformatics Institute GWAS catalog [73]. Notably, if the precise start and end positions of the locus were not provided in previous studies, we defined the boundaries by extending 1,000 kb upstream and downstream from the lead SNP to ensure a thorough comparison.

### Functional annotation and enrichment analyses

The FUMA [28] online annotation platform was employed for the functional annotation of candidate SNPs in the genetic loci. All of the SNPs were annotated with CADD scores, RegulomeDB scores, and chromatin states. The CADD score is an integrated measure of variant deleteriousness derived from 63 functional annotations, with higher scores indicating greater deleterious potential. A CADD score exceeding 12.37 was used as a threshold for potential pathogenicity [74]. RegulomeDB scores, which range from 1a to 7, were used to assess the regulatory potential of SNPs, with lower scores suggesting a higher probability of regulatory function based on evidence from expression quantitative trait loci (eQTLs) and chromatin marks [75]. Chromatin state annotations reflect the accessibility of genomic regions, assessed at 200-bp intervals and classified into 15 categorical states predicted by ChromHMM [76]. These predictions are based on 5 chromatin marks across 127 epigenomes, with states 1 to 7 representing open chromatin regions [77,78]. For each SNP, we annotated the minimum chromatin state across multiple tissue types. Next, we linked candidate SNPs to protein-coding genes using 3 gene-mapping approaches: (a) positional mapping, which links SNPs to nearby genes based on physical proximity within a 10-kb window; (b) eQTL mapping, which connects cis-eQTL SNPs to genes whose expression are influenced by allelic variation at the SNP level; and (c) chromatin interaction mapping, which associates SNPs with genes based on 3-dimensional DNA–DNA interactions between the SNP’s genomic region and nearby or distant genes. All gene-mapping analyses were restricted to brain and blood tissues, while default settings in FUMA were applied for all other parameters. To further explore the functional significance of the mapped genes, we employed the FUMA GENE2FUNC tool, which provides an analytical approach to investigating whether the gene set exhibits differential expression in different tissues or enrichment in traits from the GWAS catalog. Moreover, GO biological process enrichment analysis for all identified genes was performed using g:Profiler [79]. To account for multiple comparisons, the Benjamini–Hochberg FDR correction was applied, with a significance threshold of *q* < 0.05.

### Lifespan spatiotemporal brain expression trajectory

To investigate the expression patterns of genes shared between T2DM and subcortical brain volumes, we conducted lifespan spatiotemporal brain expression trajectory analyses utilizing processed human messenger RNA sequencing data from the PsychENCODE Consortium [29]. This dataset includes 607 tissue samples from 41 postmortem brains, covering a developmental span from 8 PCW to 40 PY. Gene expression profiles were classified into 9 developmental stages: 8 to 9 PCW, 12 to 13 PCW, 16 to 17 PCW, 19 to 22 PCW, 35 PCW to 4 months, 0.5 to 2.6 PY, 2.8 to 10.7 PY, 13 to 19 PY, and 21 to 40 PY. These stages encompass 9 anatomical tissues, including the frontal lobe, parietal lobe, temporal lobe, occipital lobe, hippocampus, amygdala, striatum, thalamus, and cerebellum. Gene expression levels for each sample were quantified by reads per kilobase of transcript per million reads mapped (RPKM). Log_2_ transformation was applied to normalize the data, and expression levels were centered around the mean [80]. The expression levels of each gene in various brain tissues were calculated by averaging RPKM values within the respective tissues, and the whole-brain expression level for each gene was determined by averaging the RPKM values across all brain tissues. For each volumetric trait, the gene expression values were subsequently calculated as the mean values of all of the shared genes mapped to their shared loci with T2DM. Ultimately, expression trajectories were fitted using a nonlinear locally estimated scatterplot smoothing regression line to capture developmental patterns across the lifespan.

### Gene-based expression–trait association analysis

We employed the MetaXcan toolkit [30], including S-PrediXcan and S-MultiXcan, to explore the associations of brain expression levels of shared genes with T2DM and subcortical volumetric traits. S-PrediXcan utilizes eQTL prediction models to estimate gene expression levels from GWAS summary statistics and evaluate the associations between predicted gene expression and the phenotypes of interest. We first applied S-PrediXcan to assess the relationship between gene expression predictions in 13 brain tissues—including the anterior cingulate cortex (BA24), frontal cortex (BA9), cortex, amygdala, caudate, hippocampus, putamen, nucleus accumbens, hypothalamus, cerebellar hemisphere, cerebellum, cervical spinal cord (C1), and substantia nigra—and T2DM and the 14 subcortical brain structures. Subsequently, we used S-MultiXcan to integrate gene expression predictions across the 13 brain tissues and jointly analyze how these combined expression patterns influence T2DM and subcortical brain structures. Associations between gene expression and traits were identified as significant with an FDR correction (*q* < 0.05).

### Validating analysis

The shared loci discovered by conjFDR analysis were validated utilizing LAVA [31], which detects local genetic relationships between complex phenotypes by identifying shared genetic signals across segmented genomic regions. Specifically, univariate tests were first conducted to estimate local genetic signals for each trait (e.g., T2DM and subcortical brain volumes) within shared loci. Loci lacking nominally significant local genetic signals were filtered out, retaining only those significantly associated with the relevant phenotypes [81]. Bivariate analyses were then performed for loci with significant local genetic signals for both traits to assess the local genetic correlation between T2DM and subcortical brain volumes. The bivariate analysis estimated the local genetic correlation coefficient (local *r*_g_) between T2DM and subcortical brain volumes, confirming the presence of shared genetic effects within these loci, with the Benjamini–Hochberg FDR correction (*q* < 0.05) was applied.

Furthermore, we investigated the consistency of allelic effect directions [82,83] for the shared top lead SNPs between the discovery and independent datasets, following the methodology used in previous studies [66,67]. For the replication analysis, we used T2DM [49] and subcortical volumetric phenotype [84] data from East Asian populations. We first selected the top lead SNPs from the discovery dataset that were located within the shared loci and then extracted the corresponding SNPs in the independent datasets. For each SNP, we compared the direction of their allelic effects between the 2 datasets to evaluate consistency. The consistency was quantified by counting the number of top lead SNPs from the discovery dataset that showed the same direction of effect in the independent datasets. To assess statistical significance, we performed an exact binomial test under the null hypothesis of a random distribution of effect directions.

## Data Availability

The GWAS meta-analysis summary statistics for T2DM were acquired from the DIAGRAM consortium (https://diagram-consortium.org/index.html). The GWAS summary statistics for subcortical brain volumes were obtained via the BIG40 web browser (https://open.win.ox.ac.uk/ukbiobank/big40/). The code used in the present study is shared on public repositories: MiXeR (https://github.com/precimed/mixer), cond/conjFDR (https://github.com/precimed/pleiofdr), FUMA (https://fuma.ctglab.nl/), g:Profiler (https://biit.cs.ut.ee/gprofiler/gost), MetaXcan (https://github.com/hakyimlab/MetaXcan), and LAVA (https://github.com/josefin-werme/LAVA).
