# Supplementary material for: Genome-wide Pleiotropy Analysis Reveals Shared Genetic Associations between Type 2 Diabetes Mellitus and Subcortical Brain Volumes
Source: Research (Wash D C). 2025 May 6;8:0688. doi: 10.34133/research.0688 (PMC12053431; doi:10.34133/research.0688)
Supplement: Supplementary 1 — Figs. S1 to S36 Tables S1 to S17 [file research.0688.f1.zip › Supplementary Information.pdf]

Supplementary Figures

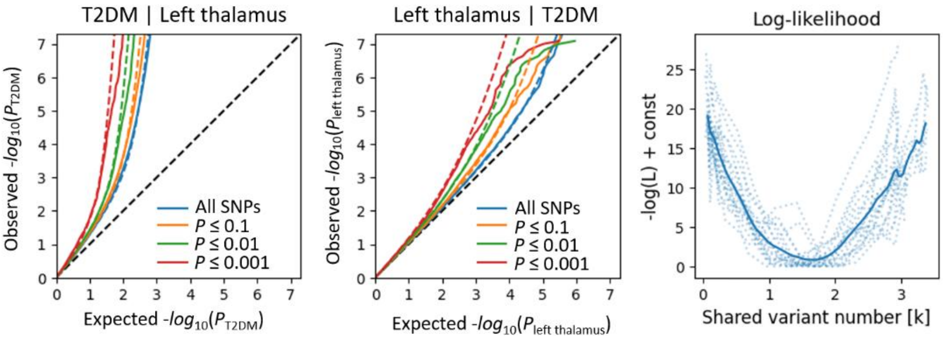

Fig. S1. The polygenic overlap between T2DM and the volume of left thalamus. The conditional *Q-Q* plots reveal the cross-trait SNP enrichment and the negative log-likelihood plot indicates the performance of the best model compared to the minimum and maximum models.

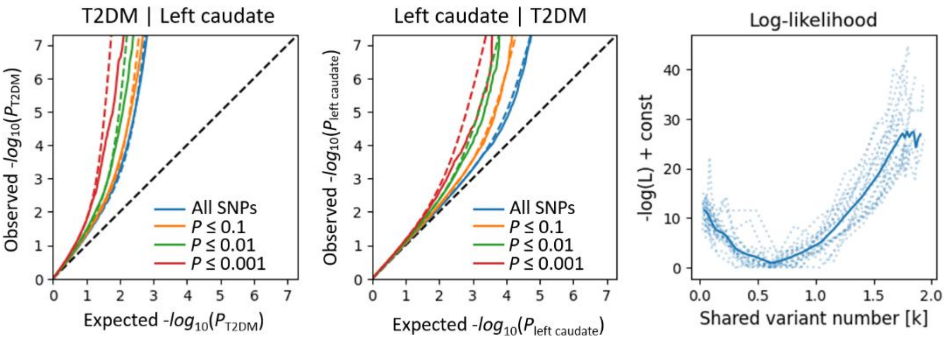

Fig. S2. The polygenic overlap between T2DM and the volume of left caudate. The conditional *Q-Q* plots reveal the cross-trait SNP enrichment and the negative log-likelihood plot indicates the performance of the best model compared to the minimum and maximum models.

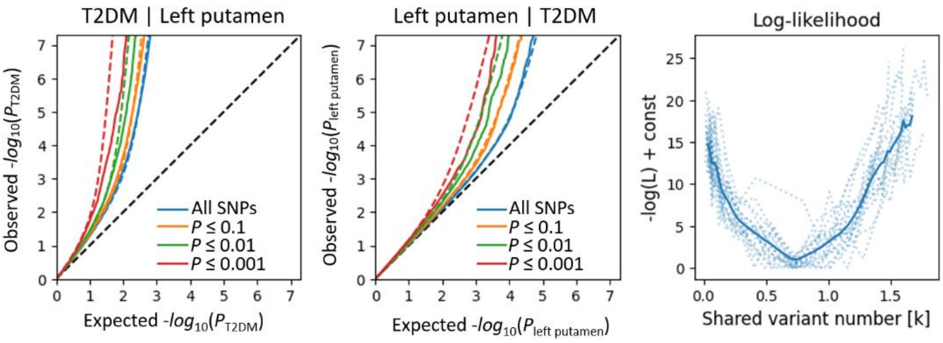

Fig. S3. The polygenic overlap between T2DM and the volume of left putamen. The conditional *Q-Q* plots reveal the cross-trait SNP enrichment and the negative log-likelihood plot indicates the performance of the best model compared to the minimum and maximum models.

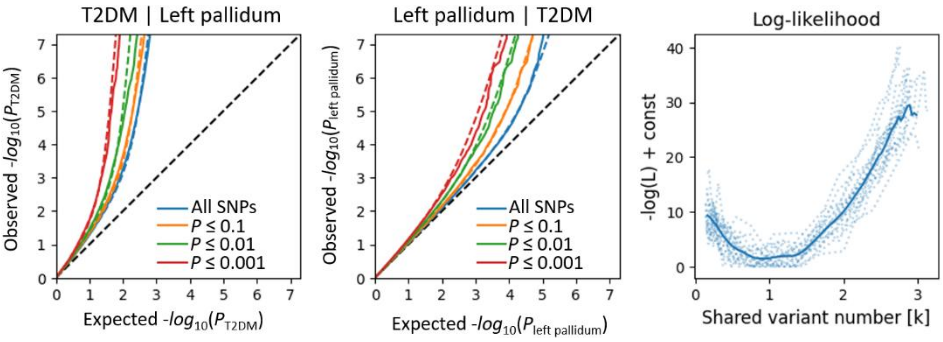

Fig. S4. The polygenic overlap between T2DM and the volume of left pallidum. The conditional *Q-Q* plots reveal the cross-trait SNP enrichment and the negative log-likelihood plot indicates the performance of the best model compared to the minimum and maximum models.

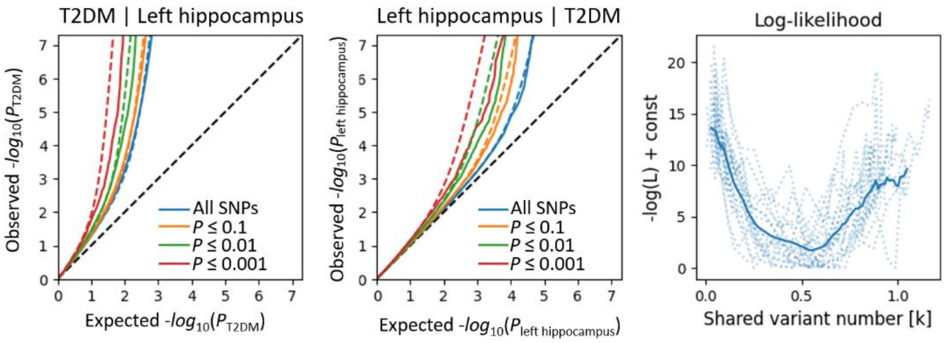

Fig. S5. The polygenic overlap between T2DM and the volume of left hippocampus.

The conditional *Q-Q* plots reveal the cross-trait SNP enrichment and the negative log-likelihood plot indicates the performance of the best model compared to the minimum and maximum models.

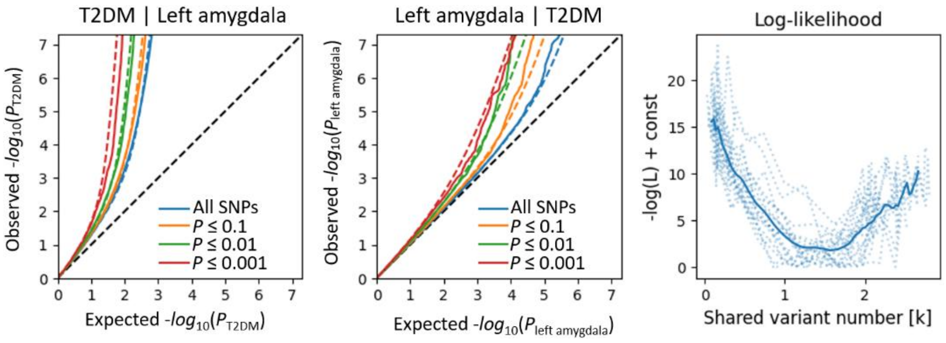

Fig. S6. The polygenic overlap between T2DM and the volume of left amygdala.

The conditional *Q-Q* plots reveal the cross-trait SNP enrichment and the negative log-likelihood plot indicates the performance of the best model compared to the minimum and maximum models.

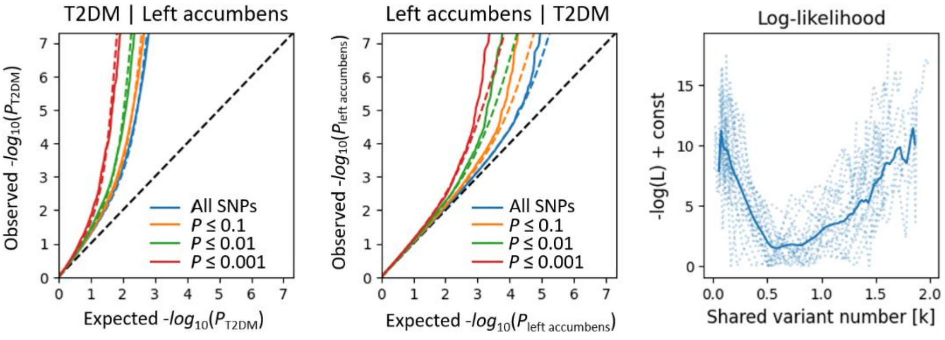

Fig. S7. The polygenic overlap between T2DM and the volume of left accumbens. The conditional *Q-Q* plots reveal the cross-trait SNP enrichment and the negative log-likelihood plot indicates the performance of the best model compared to the minimum and maximum models.

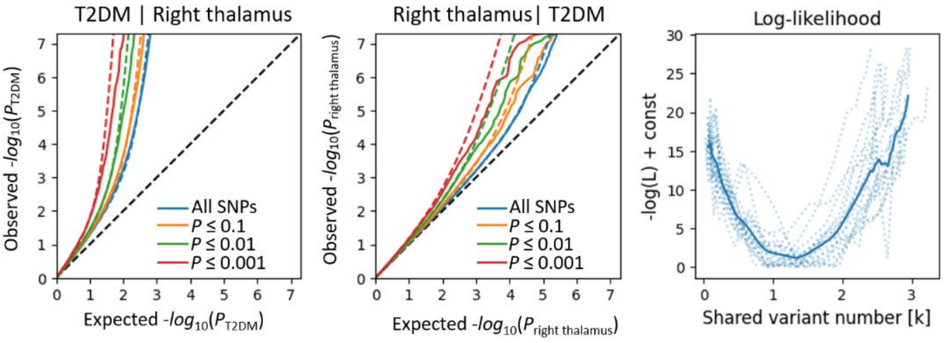

Fig. S8. The polygenic overlap between T2DM and the volume of right thalamus. The conditional *Q-Q* plots reveal the cross-trait SNP enrichment and the negative log-likelihood plot indicates the performance of the best model compared to the minimum and maximum models.

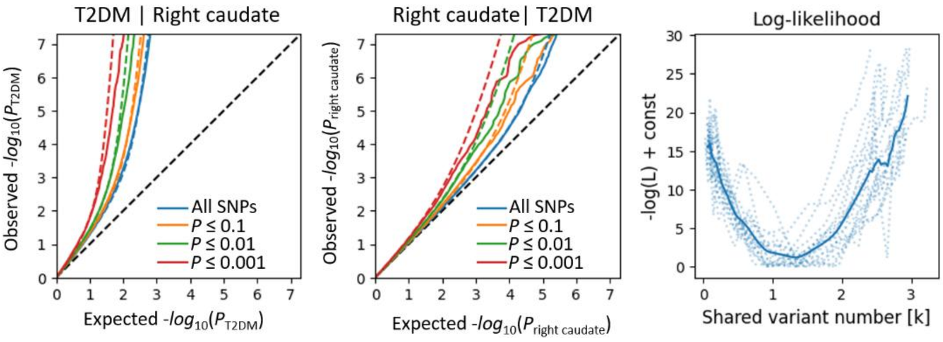

Fig. S9. The polygenic overlap between T2DM and the volume of right caudate. The conditional *Q-Q* plots reveal the cross-trait SNP enrichment and the negative log-likelihood plot indicates the performance of the best model compared to the minimum and maximum models.

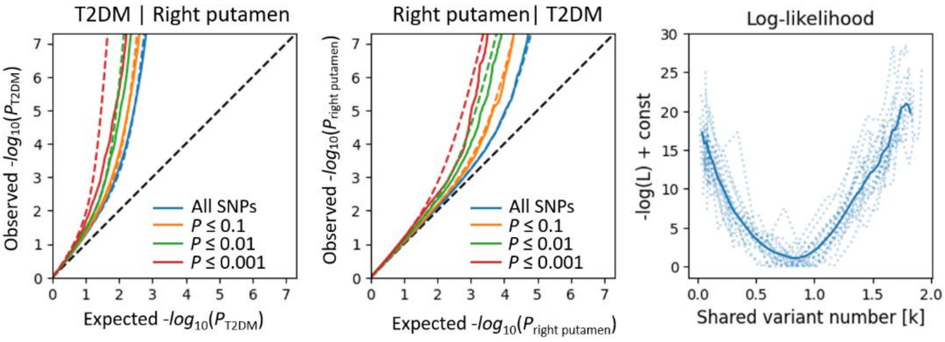

Fig. S10. The polygenic overlap between T2DM and the volume of right putamen. The conditional *Q-Q* plots reveal the cross-trait SNP enrichment and the negative log-likelihood plot indicates the performance of the best model compared to the minimum and maximum models.

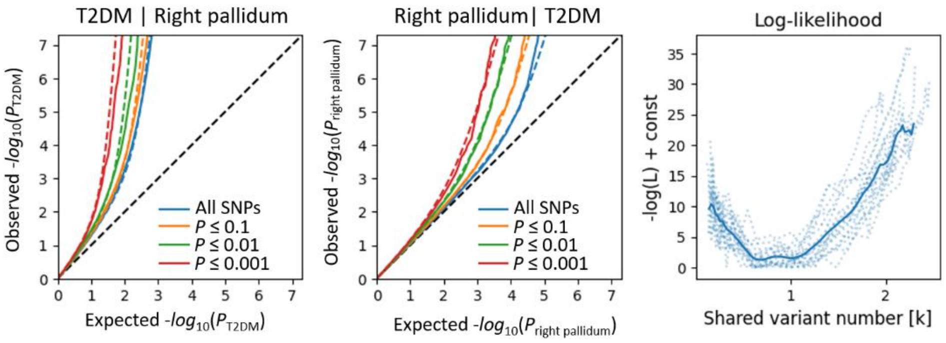

Fig. S11. The polygenic overlap between T2DM and the volume of right pallidum. The conditional *Q-Q* plots reveal the cross-trait SNP enrichment and the negative log-likelihood plot indicates the performance of the best model compared to the minimum and maximum models.

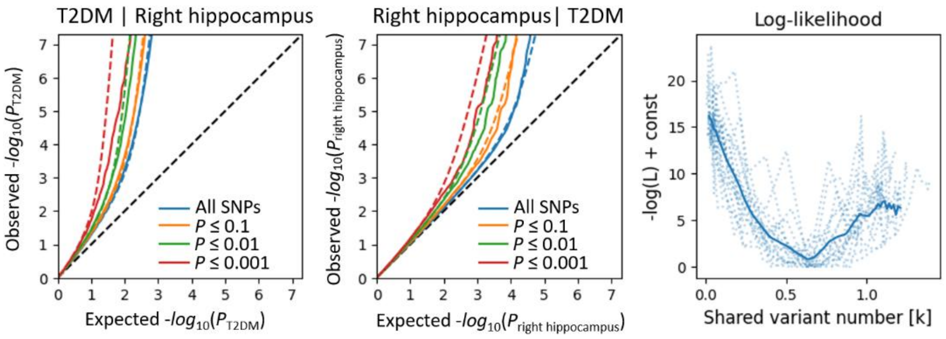

Fig. S12. The polygenic overlap between T2DM and the volume of right hippocampus. The conditional *Q-Q* plots reveal the cross-trait SNP enrichment and the negative log-likelihood plot indicates the performance of the best model compared to the minimum and maximum models.

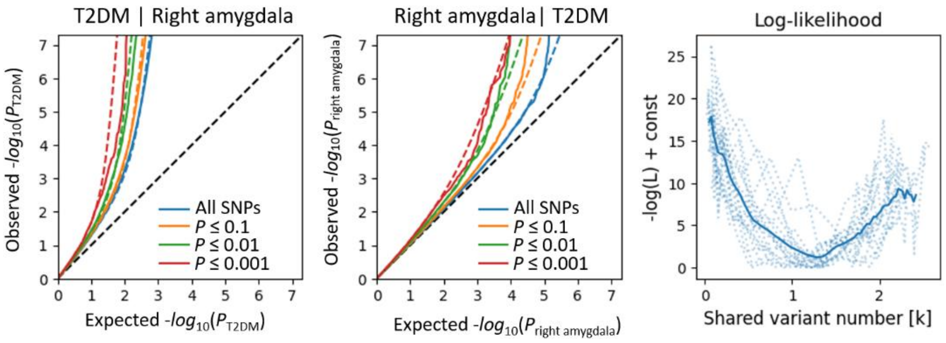

Fig. S13. The polygenic overlap between T2DM and the volume of right amygdala. The conditional *Q-Q* plots reveal the cross-trait SNP enrichment and the negative log-likelihood plot indicates the performance of the best model compared to the minimum and maximum models.

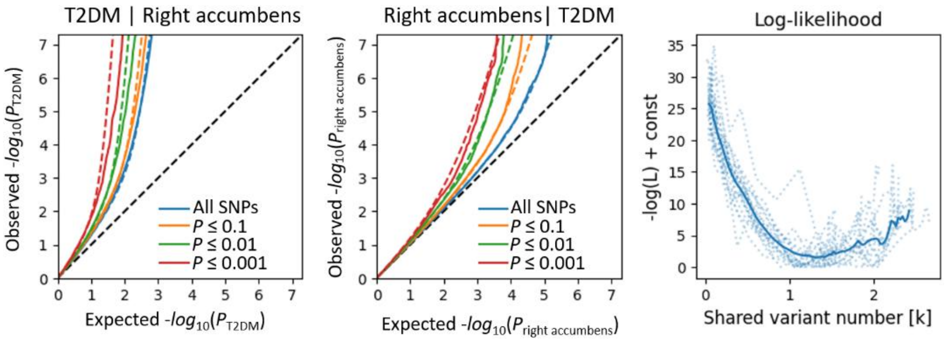

Fig. S14. The polygenic overlap between T2DM and the volume of right accumbens. The conditional *Q-Q* plots reveal the cross-trait SNP enrichment and the negative log-likelihood plot indicates the performance of the best model compared to the minimum and maximum models.

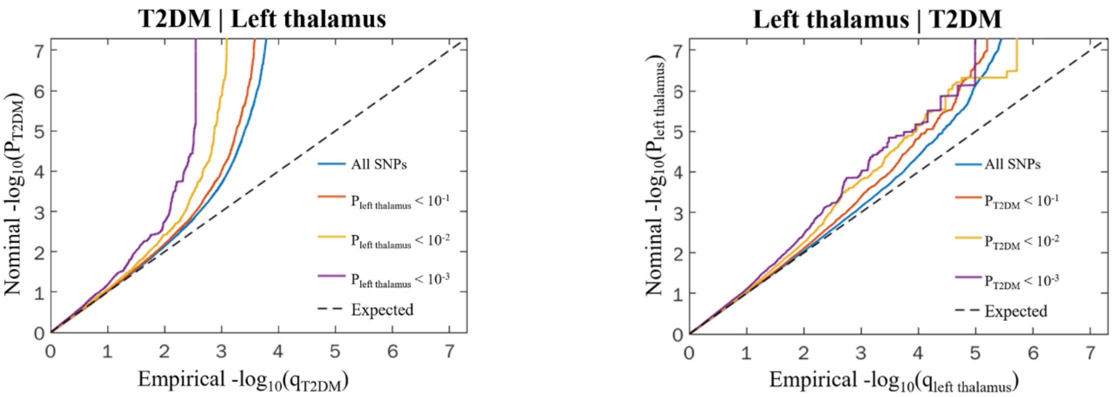

Fig. S15. Conditional *Q-Q* plots for T2DM conditioned on the volume of left thalamus and vice versa.

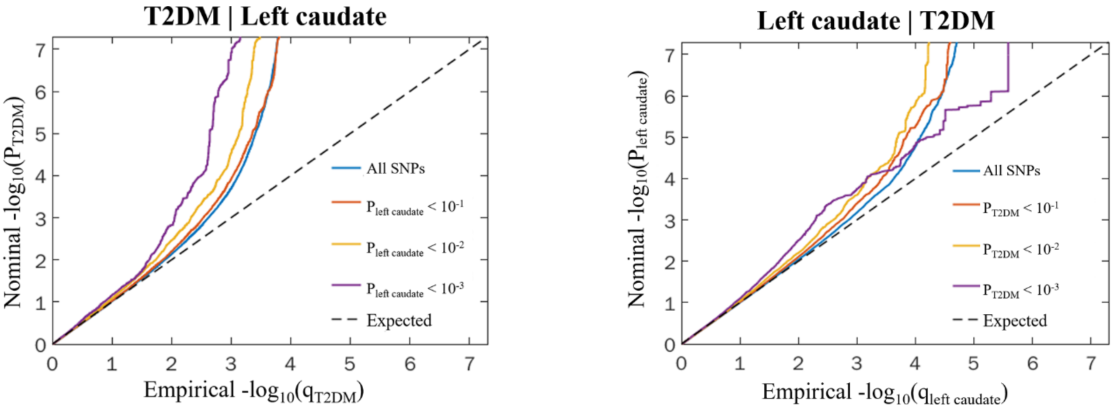

Fig. S16. Conditional *Q-Q* plots for T2DM conditioned on the volume of left caudate and vice versa.

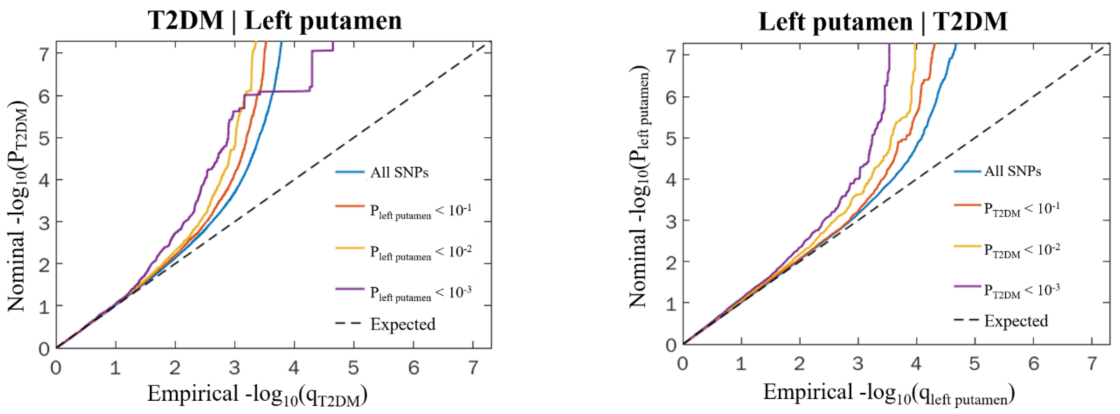

Fig. S17. Conditional *Q-Q* plots for T2DM conditioned on the volume of left putamen and vice versa.

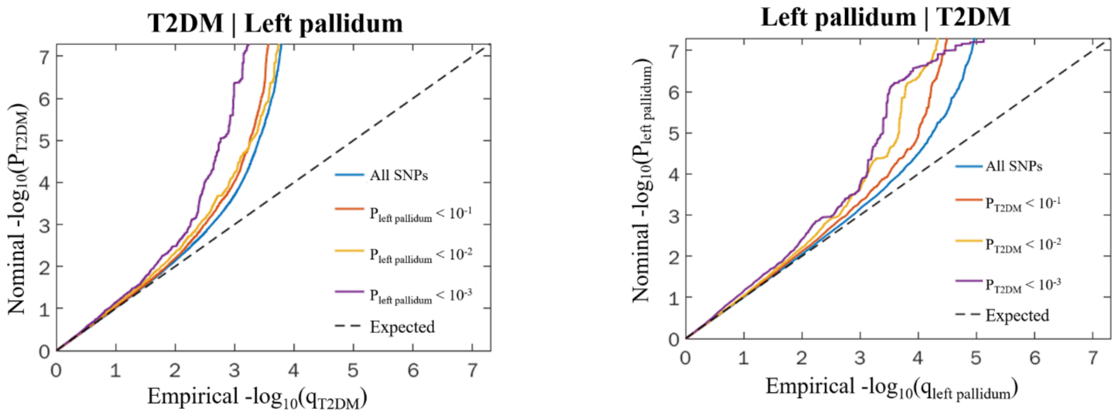

Fig. S18. Conditional *Q-Q* plots for T2DM conditioned on the volume of left pallidum and vice versa.

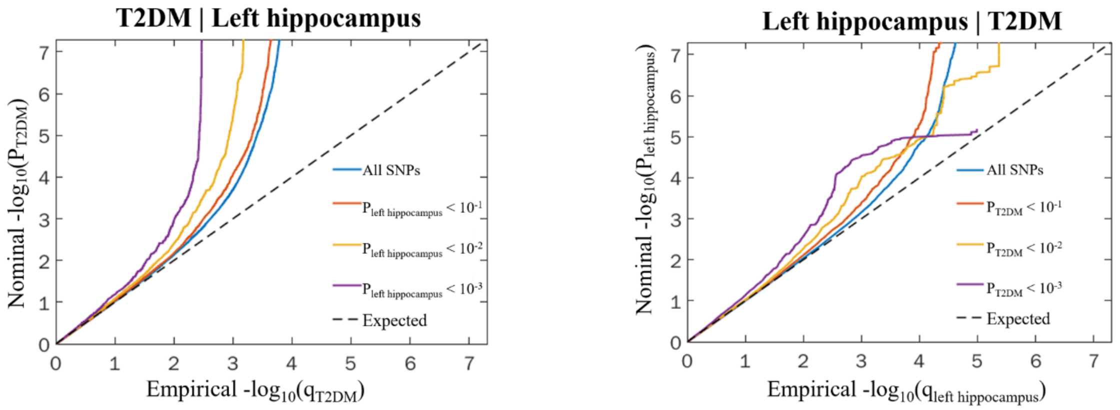

Fig. S19. Conditional *Q-Q* plots for T2DM conditioned on the volume of left hippocampus and vice versa.

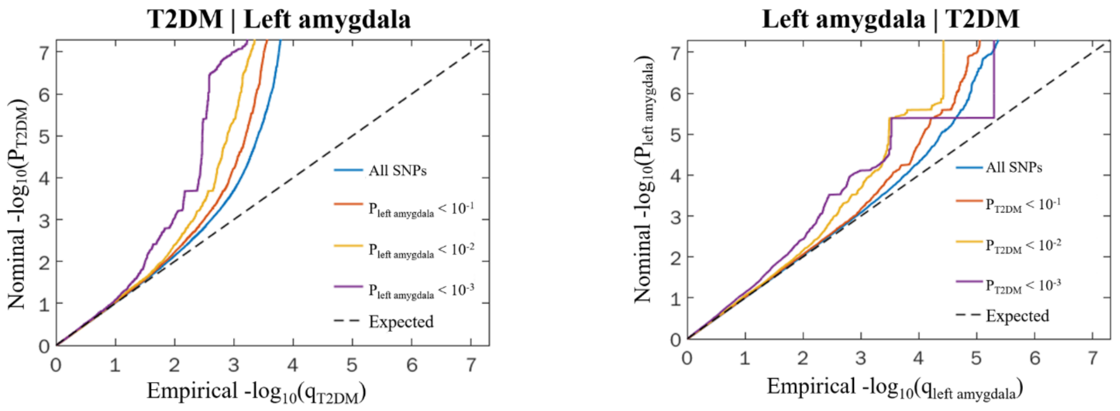

Fig. S20. Conditional *Q-Q* plots for T2DM conditioned on the volume of left amygdala and vice versa.

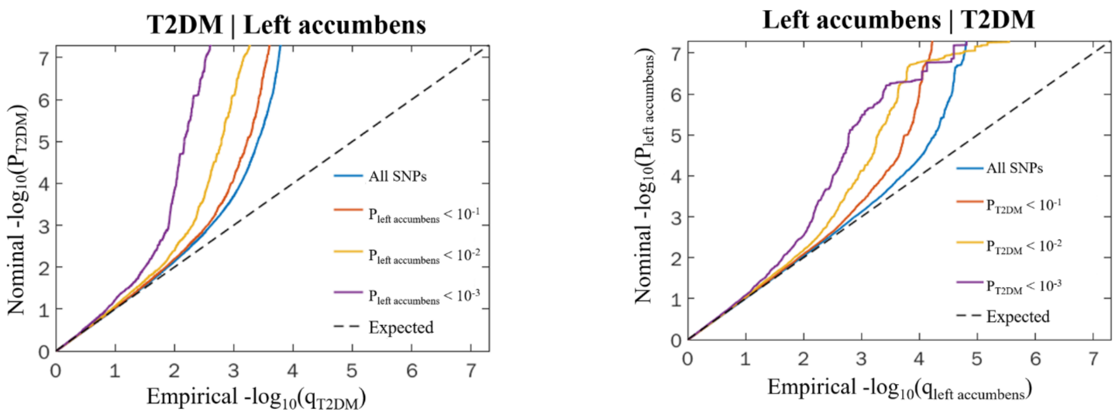

Fig. S21. Conditional *Q-Q* plots for T2DM conditioned on the volume of left accumbens and vice versa.

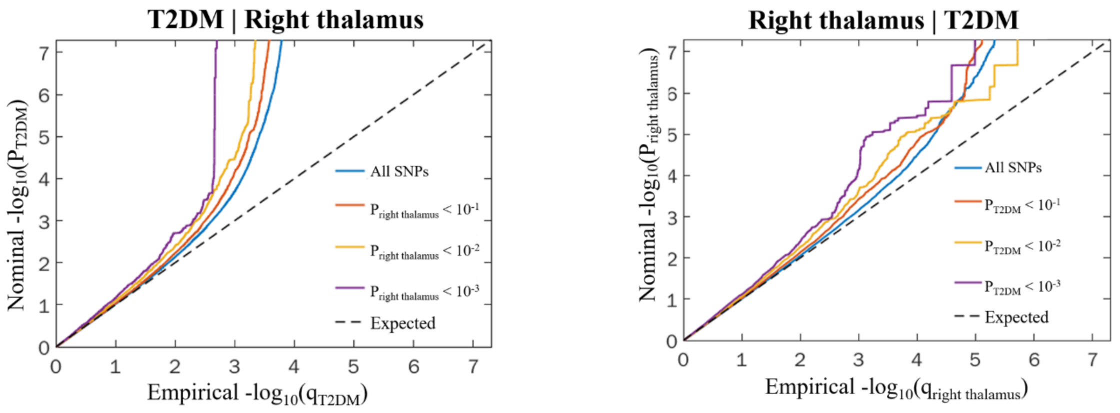

Fig. S22. Conditional *Q-Q* plots for T2DM conditioned on the volume of right thalamus and vice versa.

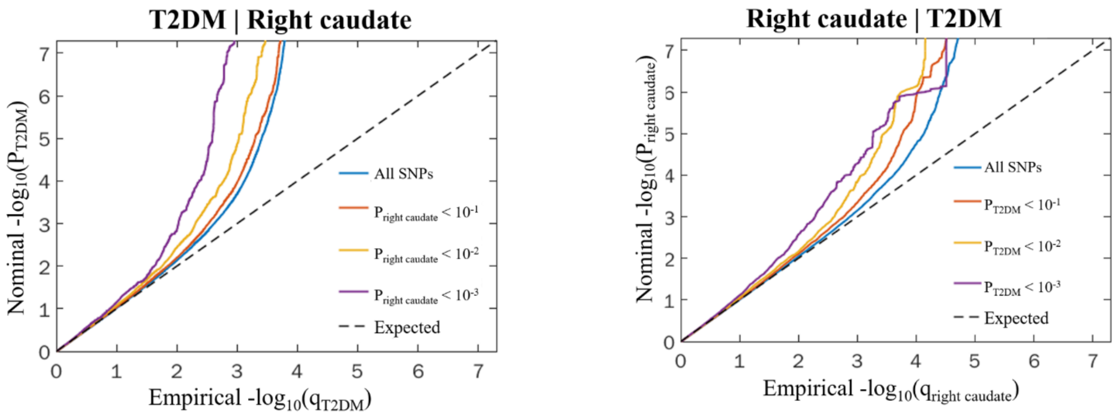

Fig. S23. Conditional *Q-Q* plots for T2DM conditioned on the volume of right caudate and vice versa.

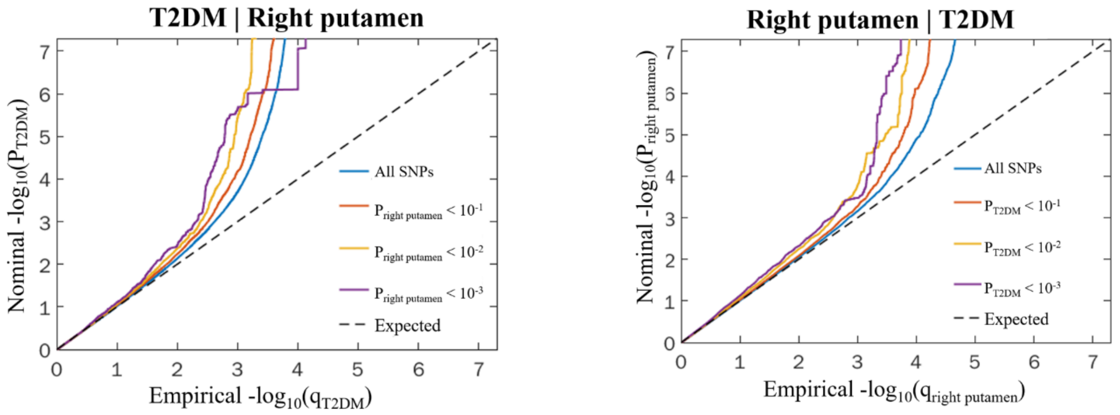

Fig. S24. Conditional *Q-Q* plots for T2DM conditioned on the volume of right putamen and vice versa.

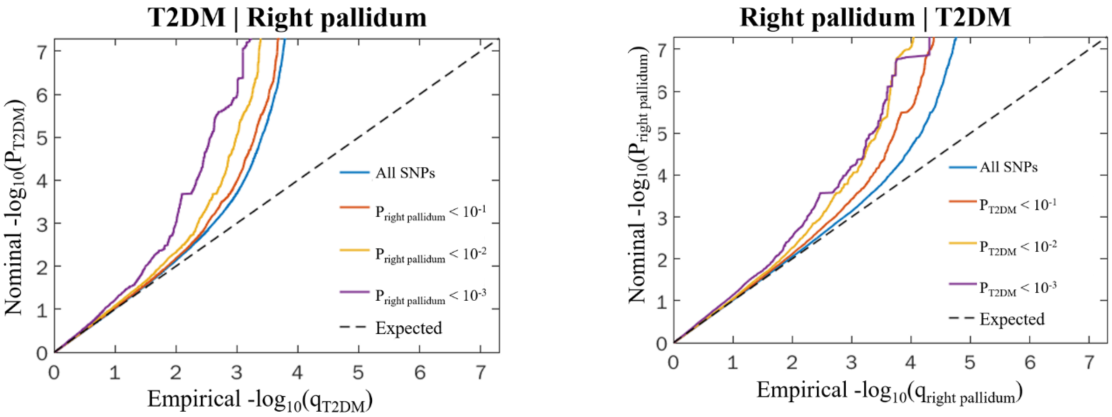

Fig. S25. Conditional *Q-Q* plots for T2DM conditioned on the volume of right pallidum and vice versa.

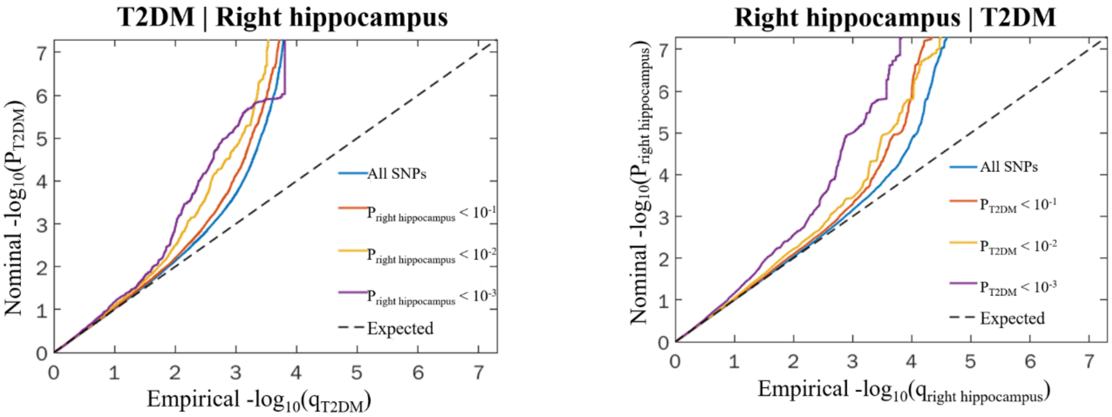

Fig. S26. Conditional *Q-Q* plots for T2DM conditioned on the volume of right hippocampus and vice versa.

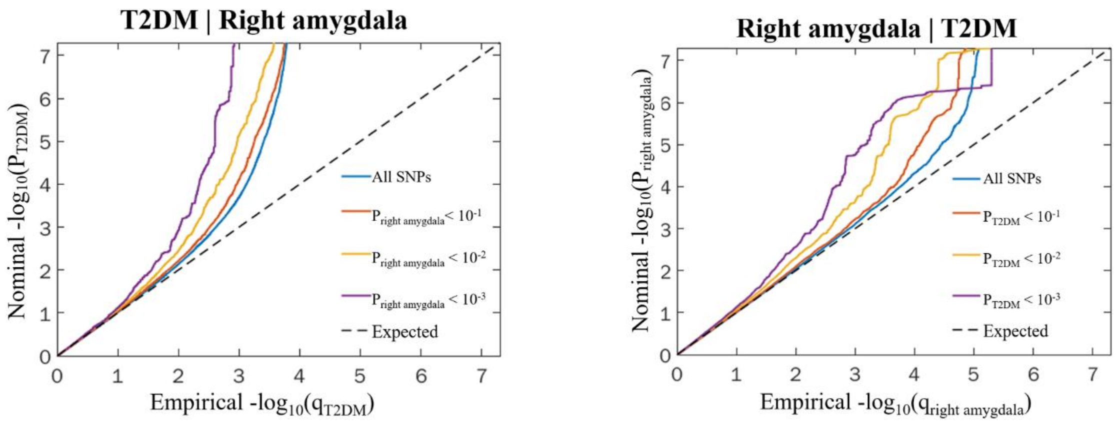

Fig. S27. Conditional *Q-Q* plots for T2DM conditioned on the volume of right amygdala and vice versa.

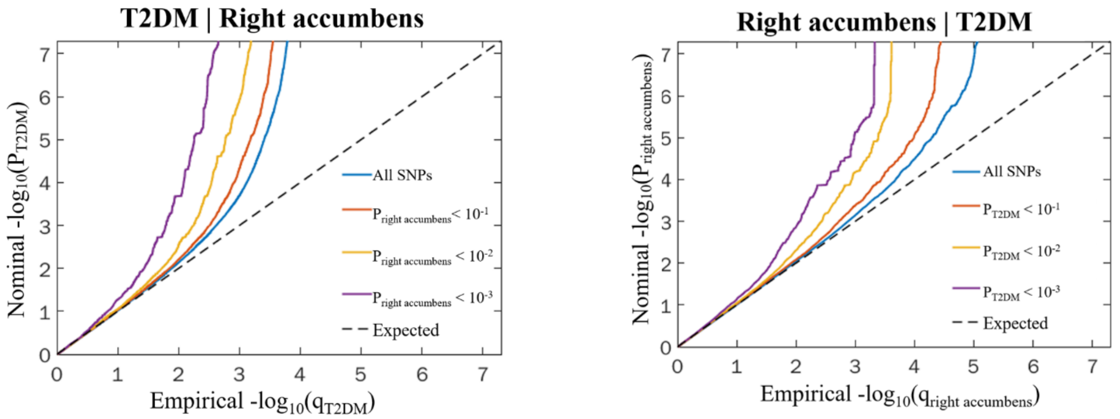

Fig. S28. Conditional *Q-Q* plots for T2DM conditioned on the volume of right accumbens and vice versa.

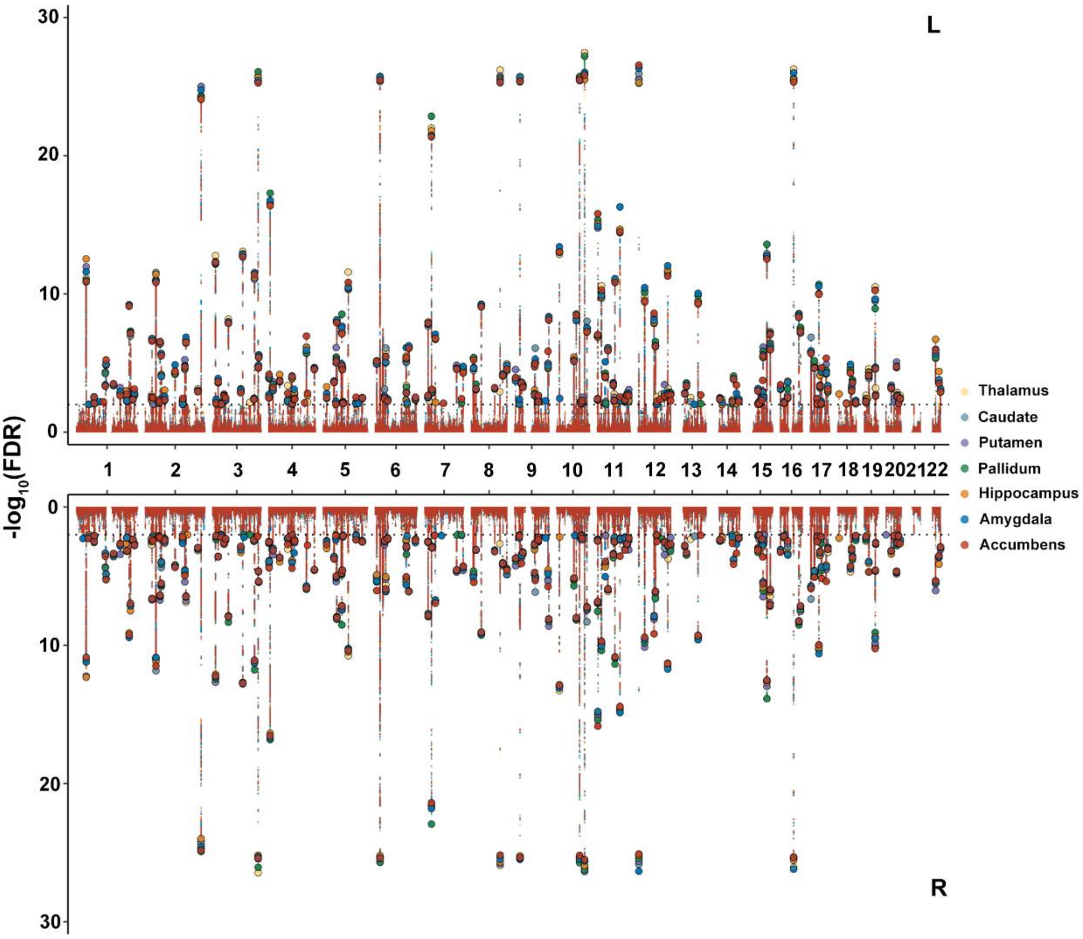

Fig. S29. Manhattan plot showing the genetic associations for T2DM conditioned on each subcortical volumetric trait. The top and bottom panel show results of T2DM conditioned on left and right subcortical volumetric traits, respectively. Each point represents an SNP, with larger points outlined in black indicating lead SNPs. The *y*-axis shows -$\log_{10}$-transformed condFDR values for each SNP, and the *x*-axis indicates the chromosomal position. The threshold of condFDR = 0.01 is represented by the dashed line, with different subcortical volumetric traits indicated by the color of the dots. **Abbreviations:** L, left; R, right.

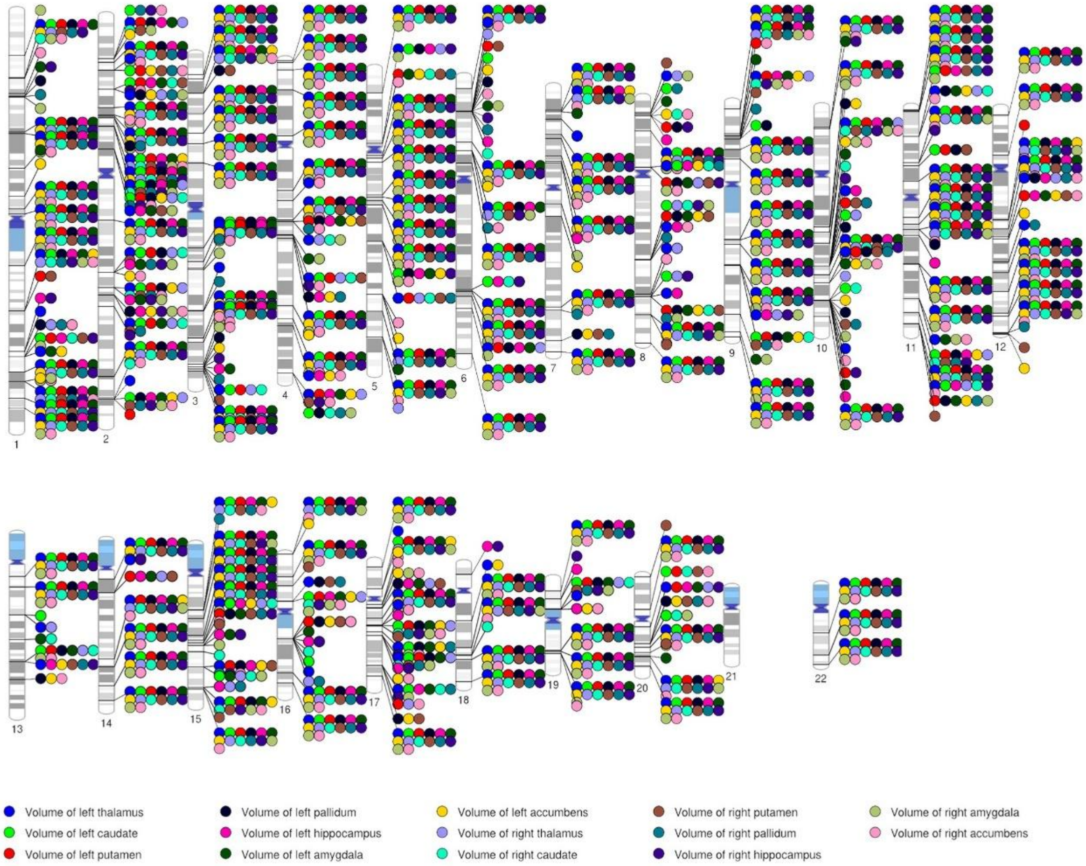

Fig. S30. Conditional FDR results show the distribution of top lead SNPs for T2DM, conditioned on each subcortical volumetric trait, across the genomic landscape of chromosomes. Different colors correspond to each subcortical volumetric phenotype, as indicated in the legend.

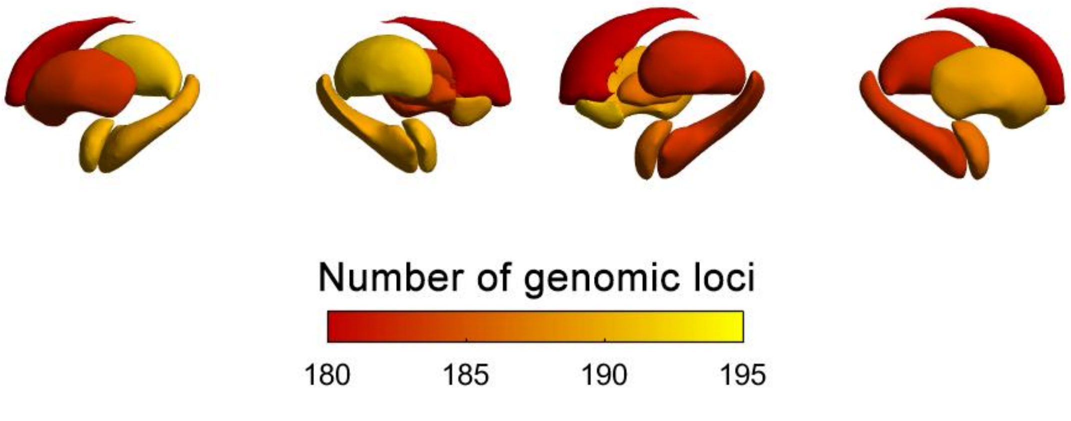

Fig. S31. Brain map displaying the locus count for T2DM conditioned on subcortical volumetric traits. As the color shifts from red to yellow, it signifies a growing number of genomic loci.

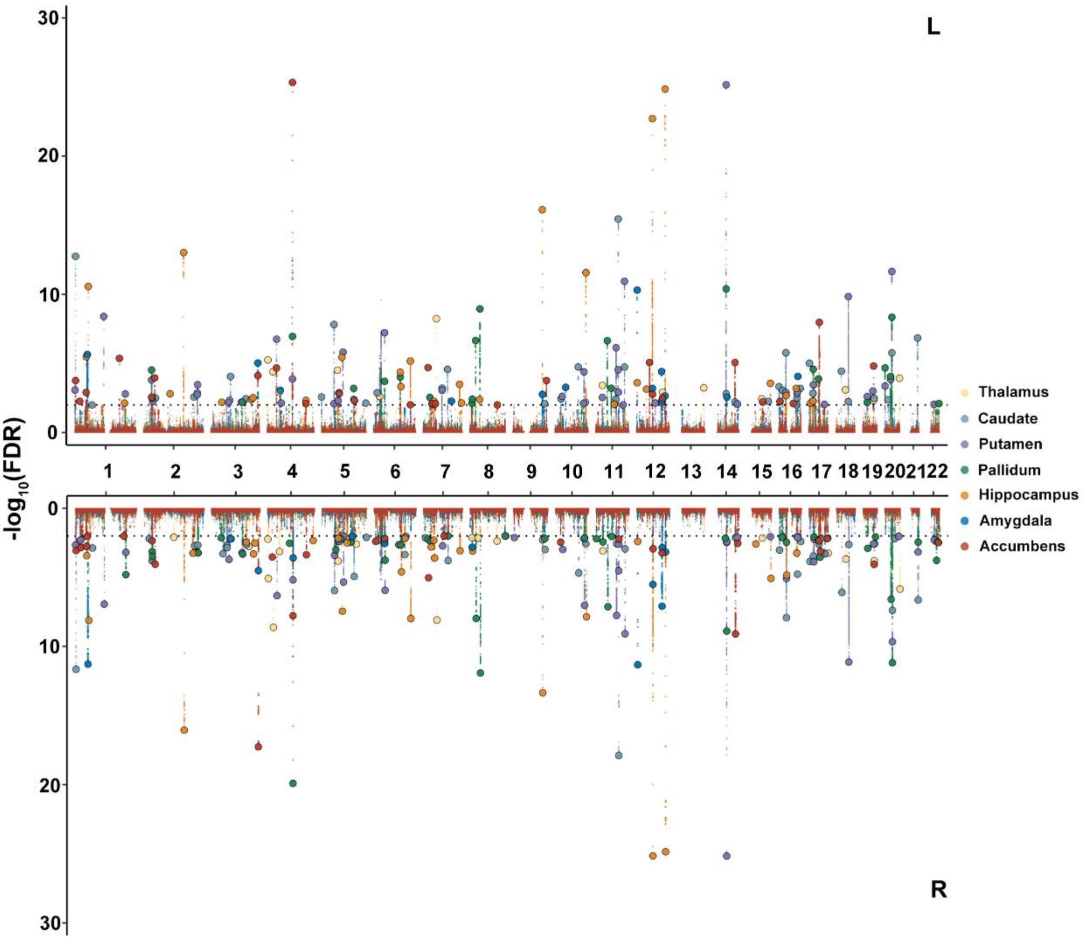

Fig. S32. Manhattan plot showing the genetic associations for each subcortical volumetric trait conditioned on T2DM. The top and bottom panels show results of left and right subcortical volumetric traits, respectively. Each point represents an SNP, with larger points outlined in black indicating lead SNPs. The *y*-axis shows -log$_{10}$-transformed condFDR values for each SNP, and the *x*-axis indicates the chromosomal position. The threshold of condFDR = 0.01 is represented by the dashed line, with different subcortical volumetric traits indicated by the color of the dots. **Abbreviations:** L, left; R, right.

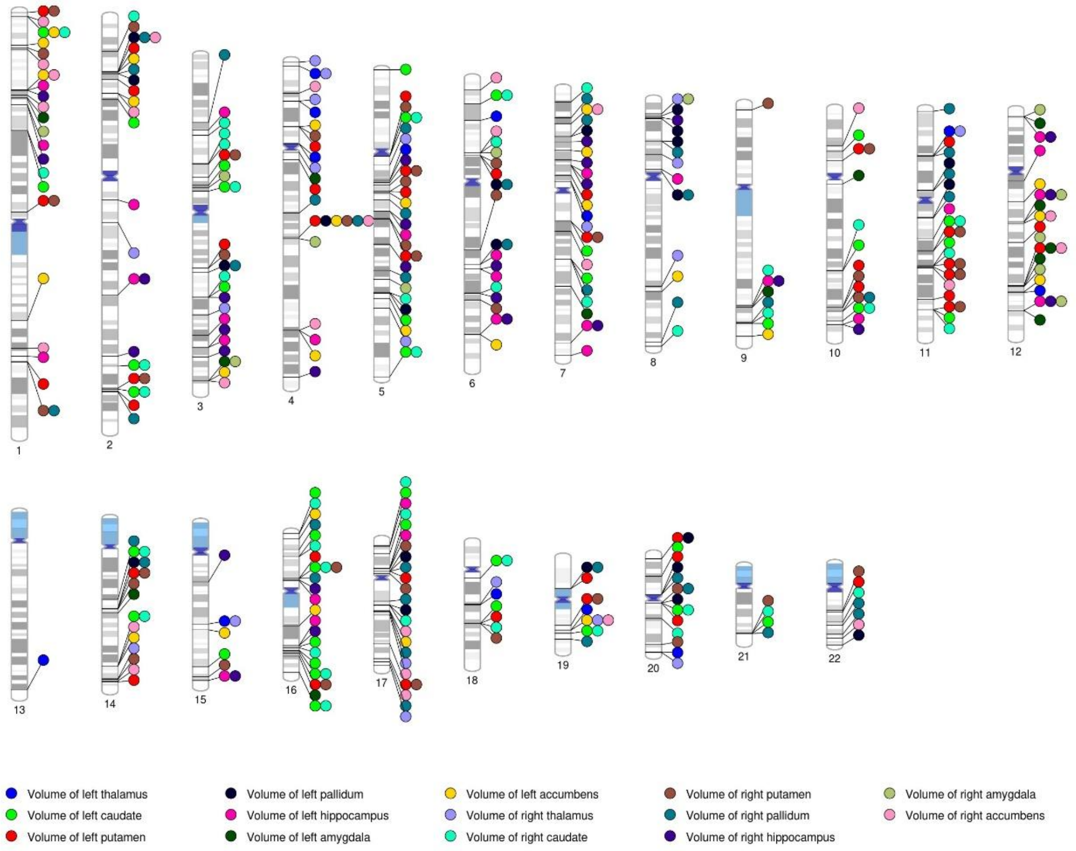

Fig. S33. Conditional FDR results show the distribution of top lead SNPs for each subcortical volumetric trait, conditioned on T2DM, across the genomic landscape of chromosomes. Different colors correspond to each subcortical volumetric phenotype, as indicated in the legend.

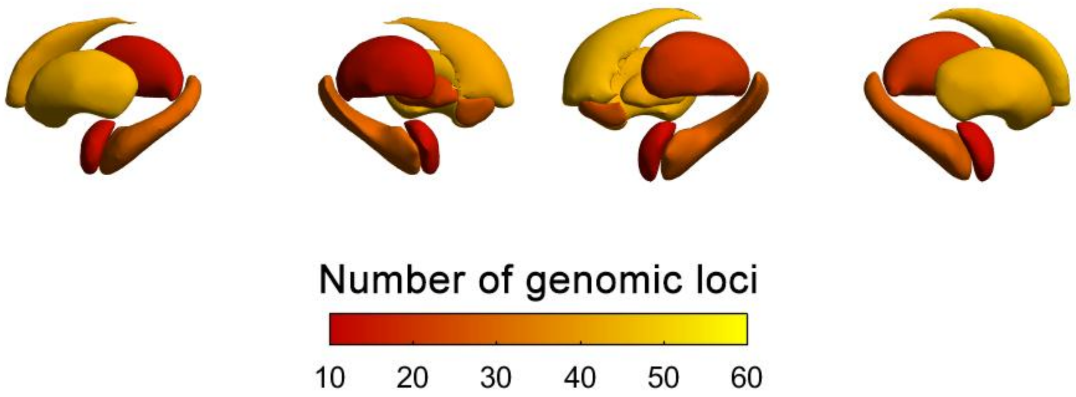

Fig. S34. Brain map displaying the locus count for subcortical volumetric traits conditioned on T2DM. As the color shifts from red to yellow, it signifies a growing number of genomic loci.

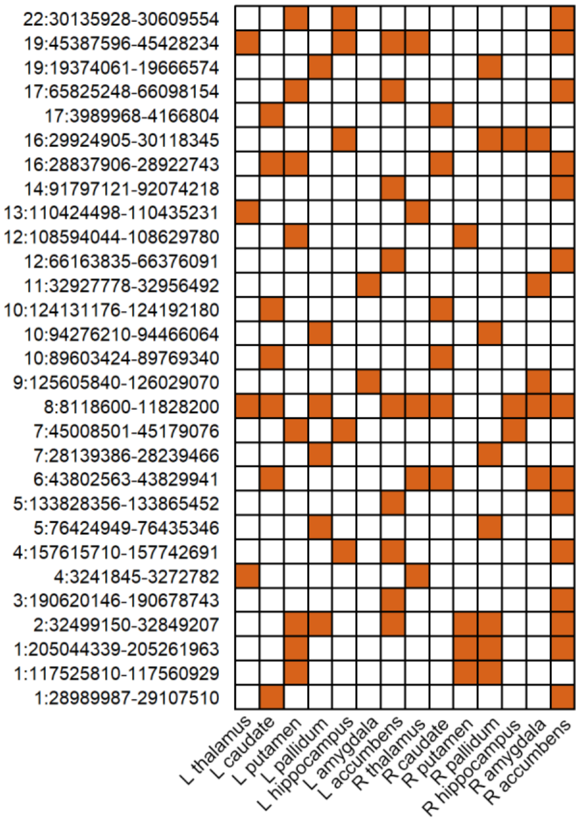

Fig. S35. Matrix plot of overlapping genomic loci associated with T2DM and at least two subcortical volumetric traits. The plot shows 29 overlapping genomic loci associated with T2DM and at least two subcortical volumetric traits at conjFDR < 0.05. Each orange square represents a genomic locus associated with both T2DM and the corresponding subcortical volumetric trait, and each white square indicates a genomic locus that is not associated with the trait. **Abbreviations:** L, left; R, right.

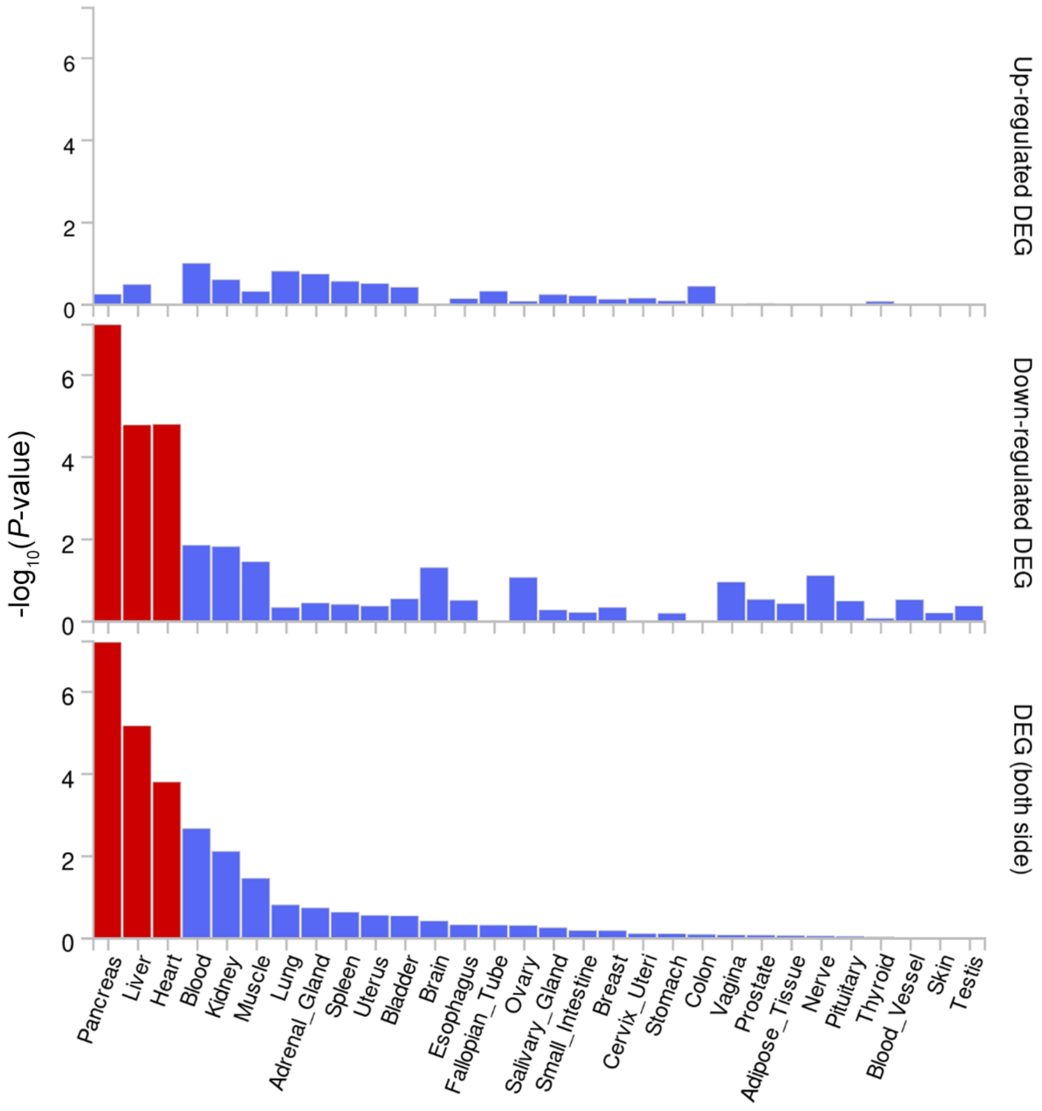

Fig. S36. Histogram showing whether the gene set associated with T2DM and subcortical volumetric traits is a differential expression of gene set (DEG) in different tissues. Red bars represent statistical significance after adjusting for multiple comparisons. The histogram illustrates tissue specificity across 30 tissue types from GTEx v8.
